# Large Language Model Adaptation Strategies in Speech-Based Cognitive Screening: Systematic Evaluation

**DOI:** 10.2196/82608

**Published:** 2026-03-26

**Authors:** Fatemeh Taherinezhad, Mohamad Javad Momeni Nezhad, Sepehr Karimi, Sina Rashidi, Ali Zolnour, Maryam Dadkhah, Yasaman Haghbin, Hossein Azadmaleki, Maryam Zolnoori

**Affiliations:** 1 Columbia University Irving Medical Center, 622 W, 168th St, New York, NY, 10032, United States; 2 School of Nursing, Columbia University, 560 W, 168th St, New York, NY, 10032, United States, 1 212-305-5756; 3 Data Science Institute, Columbia University, New York, NY, United States

**Keywords:** cognitive impairment detection, speech-based screening, large language models adaptation, in-context learning, reasoning-augmented prompting, fine-tuning, multimodal speech-text analysis

## Abstract

**Background:**

Over half of US adults with Alzheimer disease and related dementias (ADRD) remain undiagnosed. Speech-based screening algorithms offer a scalable approach, but the relative value of large language model (LLM) adaptation strategies is unclear.

**Objective:**

The study aimed to compare LLM adaptation strategies for cognitive impairment detection across DementiaBank speech datasets using both text-only and multimodal models.

**Methods:**

We analyzed audio-recorded speech from 237 participants in the ADReSSo subset of DementiaBank (ADRD vs cognitive normal [CN]) and report performance on a held-out test set (n=71). Nine text-only LLMs (3B-405B; open-weight and commercial) and 3 multimodal audio-text models were evaluated. Adaptations included (1) in-context learning (ICL) with 4 demonstration selection strategies (most similar, least similar, average similar or prototype, and random), (2) reasoning-augmented prompting (self- or teacher-generated rationales, self-consistency, tree-of-thought with domain experts), (3) parameter-efficient fine-tuning (token-level vs added classification head), and (4) multimodal audio-text integration. Generalizability of the adaptation strategies was evaluated on the DementiaBank Delaware dataset (n=205; mild cognitive impairment vs CN) using the first 3 strategies. The primary outcome was the *F*_1_-score for the cognitive impaired class; the area under the receiver operating characteristic curve was reported when available.

**Results:**

On the ADReSSo dataset, average similar (prototype) demonstrations achieved the highest ICL performance across model sizes (*F*_1_-score up to 0.81). Reasoning primarily benefited smaller models: teacher-generated rationales increased LLaMA 8B from *F*_1_-score 0.72 to 0.76; expert-role tree-of-thought improved its zero-shot score from 0.65 to 0.71. Token-level fine-tuning produced the highest scores (LLaMA 3B: *F*_1_=0.83, 95% CI 0.01, area under the curve [AUC]=0.91; LLaMA 70B: *F*_1_=0.82, 95% CI 0.02, AUC=0.86; GPT-4o: *F*_1_=0.79, 95% CI 0.01, AUC=0.87). A classification head markedly improved MedAlpaca 7B (*F*_1_=0.06, 95% CI 0.02 to *F*_1_=0.81, 95% CI 0.04), indicating model-dependent benefits of this approach. Among multimodal models, fine-tuned Phi-4 Multimodal reached an *F*_1_-score of 0.80 (cognitive impaired) and 0.75 (CN) but did not exceed the top text-only systems. On the Delaware dataset, ICL achieved a high performance (LLaMA 8B: *F*_1_=0.74; GPT-4o: *F*_1_=0.80). Reasoning-augmented ICL improved LLaMA 8B to an *F*_1_-score of 0.75. Token-level fine-tuning produced the highest scores (LLaMA 8B: *F*_1_=0.76, 95% CI 0.02; GPT-4o: *F*_1_=0.82, 95% CI 0.03).

**Conclusions:**

Detection accuracy is influenced by demonstration selection, reasoning design, and tuning method. Token-level fine-tuning is generally most effective, while a classification head benefits models that perform poorly under token-based supervision. Properly adapted open-weight models can match or exceed commercial LLMs, supporting their use in scalable speech-based ADRD and mild cognitive impairment screening. Current multimodal models may require improved audio-text alignment and/or larger training corpora.

## Introduction

Alzheimer disease (AD) and related dementias (ADRD) pose a significant public health challenge, currently affecting approximately 5 million individuals, or 11% of older adults in the United States [1-3]. This number is projected to rise to 13.2 million by 2050 [4], underscoring the need for early, scalable detection strategies. Despite national efforts, over half of individuals with ADRD remain undiagnosed and untreated [5-7]. To address this gap, the National Institute on Aging has prioritized the development of accurate, accessible screening tools [7,8].

A promising direction involves natural language processing to analyze spontaneous speech, which may reveal subtle cognitive changes missed by conventional screening [9]. Picture description tasks, such as the “Cookie Theft” scene [10], are widely used to elicit language markers of early decline. Prior pipelines follow two main approaches: (1) engineering acoustic and linguistic features [11-13] (eg, lexical diversity and syntactic complexity), and (2) fine-tuning transformer encoders such as Bidirectional Encoder Representations from Transformer (BERT) [14] (for transcripts) and Wav2Vec 2.0 [15] (for raw audio). While both strategies show promise, they require extensive feature engineering and large labeled corpora [16-18]—resources often lacking in clinical settings—limiting generalizability across dialects and institutions [19].

Large language models (LLMs) offer new opportunities for cognitive impairment detection by modeling complex linguistic patterns, performing few-shot in-context learning (ICL) [20], generating reasoning chains, and adapting via fine-tuning. LLMs show strong performance in clinical decision support tasks [21-25], including detection of depression [26], anxiety [27], suicide risk [28], and medication-related errors [29]. Applications to cognitive impairment are emerging but remain limited—for example, using GPT-4 in zero-shot fluency scoring, GPT-3 embeddings for classification, or comparing GPT-3.5, GPT-4, and Bard [30] on DementiaBank [31] transcripts. These studies suggest feasibility but lack systematic comparisons of prompting methods, fine-tuning, and multimodal inputs.

**Figure 1.**
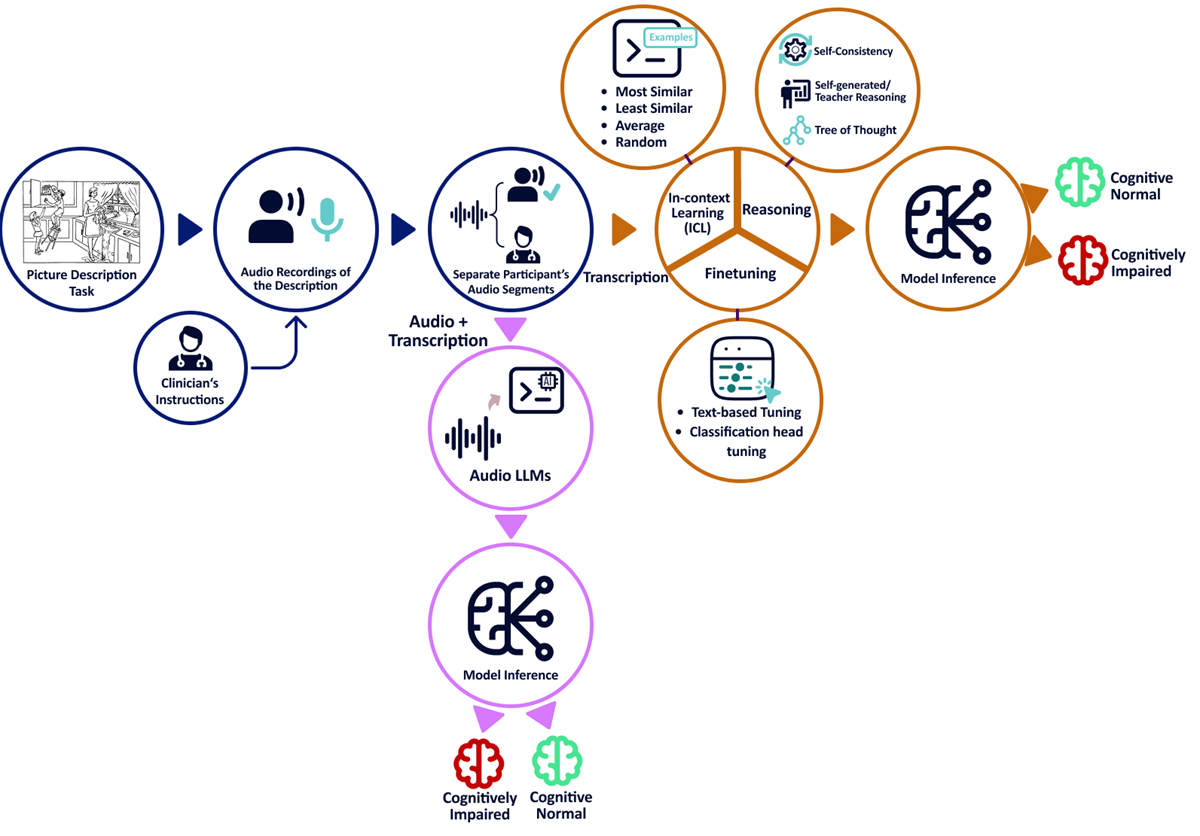
Study workflow and evaluation framework for LLM-based ADRD detection. Participants complete the Cookie-Theft picture-description task, and their responses are audio-recorded under standardized clinician instructions. Recordings are segmented per speaker and transcribed with AWS. Text-only (transcription) pipeline (orange) includes: (1) In-context learning (ICL) with demonstration selection—few-shot examples are drawn from cognitively normal (CN) and cognitively impaired (CI) speakers using four sampling rules (Most Similar, Least Similar, Average Similar, Random); (2) Reasoning-augmented prompting models receive self-generated/teacher rationales, self-consistency voting, or tree-of-thought chains; (3) Parameter-efficient fine-tuning using supervised text-based tuning and addition of a lightweight classification head. Audio-enabled pipeline (purple): Raw speech and its transcript are fed to multimodal / audio LLMs, which directly encode acoustic and linguistic cues before inference, yielding the same binary outcome labels.

We present the first comprehensive evaluation of state-of-the-art LLMs, including open-weight (LLaMA [32], Ministral [33], MedAlpaca [34], DeepSeek [35]) and commercial models (GPT-4o [36], Gemini 2.0 Flash [37]), for early detection of ADRD using the ADReSSo dataset from DementiaBank [38]. Our study comprises four components (Figure 1): (1) ICL with demonstration selection to assess the impact of different sampling strategies; (2) reasoning-augmented prompting to evaluate whether structured reasoning enhances LLM performance, particularly in smaller models; (3) parameter-efficient fine-tuning to improve classification accuracy beyond prompt-based methods; and (4) evaluation of multimodal LLMs that integrate audio and text to determine the added value of acoustic information. To assess generalizability beyond a single dataset and task, we additionally evaluate 3 components for mild cognitive impairment (MCI) detection on the DementiaBank Delaware dataset [39], which includes multiple speech tasks.

## Methods

### Dataset

This study analyzed audio recordings from the ADReSSo dataset, a subset of DementiaBank [31] Pitt Corpus picture-description task (Table 1). The dataset contains 237 participants—122 cognitive impaired and 115 cognitive normal (CN). Following ADReSSo’s original split, 166 participants (n=87 cognitive impaired and n=79 CN) formed the development set, and 71 (n=35 cognitive impaired and n=36 CN) constituted the held-out test set. All diagnoses were made by neurologists or certified cognitive specialists.

**Table 1.** Characteristics of the participants in “the ADReSSo dataset.”

Attribute	Train		Validation		Test	
	Case	Control	Case	Control	Case	Control
Participants (n)	60	56	27	23	35	36
Gender (F/M)	39/21	37/19	19/8	15/8	21/14	23/13
Age (y), mean (SD)	69.33 (7.14)	66.27 (6.81)	70.59 (6.01)	65.48 (4.72)	68.51 (7.12)	66.11 (6.53)
Age range (y)	53‐79	54‐80	60‐80	56‐74	56‐79	56‐78
Age quartiles (y; 25%, 50%, 75%)	(65, 70, 75)	(60.75, 67, 71.25)	(65, 72, 76.5)	(63.5, 66, 68)	(63, 69, 74)	(61, 66, 70)
MMSE^a^, mean (SD)	17.80 (5.04)	29.04 (1.13)	16.63 (5.94)	28.87 (1.22)	18.86 (5.8)	28.91 (1.25)
MMSE range	7‐28	26‐30	3‐27	26‐30	5‐27	24‐30
MMSE quartiles (25%, 50%, 75%)	(14.75, 18, 20)	(28, 29, 30)	(13.5, 17, 20.5)	(28.5, 29, 30)	(16, 20, 24)	(28, 29, 30)
Recording length, mean (SD)	87.20 (48.35)	68.98 (25.85)	88.52 (43.27)	68.25 (25.43)	79.42 (36.79)	66.35 (28.17)
Recording length range	35.26‐268.49	22.79‐168.61	39.91‐219.5	26.16‐121.47	28.39‐150.15	22.35‐135.68
Recording length quartiles (25%, 50%, 75%)	(54.28, 75.93, 99.94)	(52.15, 67.6, 77.8)	(60.01, 80.24, 97.45)	(44.54, 67.77, 82.11)	(51.52, 70.20, 106.97)	(44.4, 66.04, 77.69)
Word count, mean (SD)	82.63 (43.32)	114.43 (78.21)	101.67 (55.49)	111.39 (43.18)	92.49 (57.38)	111.72 (53.86)
Word count range	22‐189	21‐523	31‐284	54‐197	27‐256	45‐243
Word count quartiles (25%, 50%, 75%)	(51.25, 70.5, 106.25)	(67.25, 101, 139.75)	(67, 93, 118)	(78.5, 91, 147)	(50, 70, 120.5)	(63.5, 97, 168.25)

a MMSE: Mini-Mental State Examination.

A validation set was drawn from the development data via stratified sampling on diagnosis, Mini-Mental State Examination (MMSE) score, gender, and audio duration, yielding 116 training and 50 validation subjects. Recordings were transcribed with Amazon Web Services (AWS) General Transcribe [40].

According to the ADReSSo organizers, each audio file is a description of the “Cookie Theft” picture from the Boston Diagnostic Aphasia Exam, recorded at a 16 kHz sampling rate. The preprocessing steps included: after speaker diarization (patient-clinician), the clinician’s speech was removed; and (2) noise reduction was performed using spectral subtraction and amplitude normalization.

We denote the transcription of a subject i in $S_{i}^{C}$, in which S represents the subject and the superscript C indicates the subject’s cognitive status, with C∈{CI,CN}.

Participants were aged 53 years or older; women comprised more than 60% of each group. MMSE scores ranged from 3‐28 in cognitive impaired (mild-severe impairment) and greater than 24 in CN. CN speakers produced more words on average, whereas cognitive impaired speakers had longer recordings, suggesting slower speech or greater effort (Table 1).

**Figure 2.**
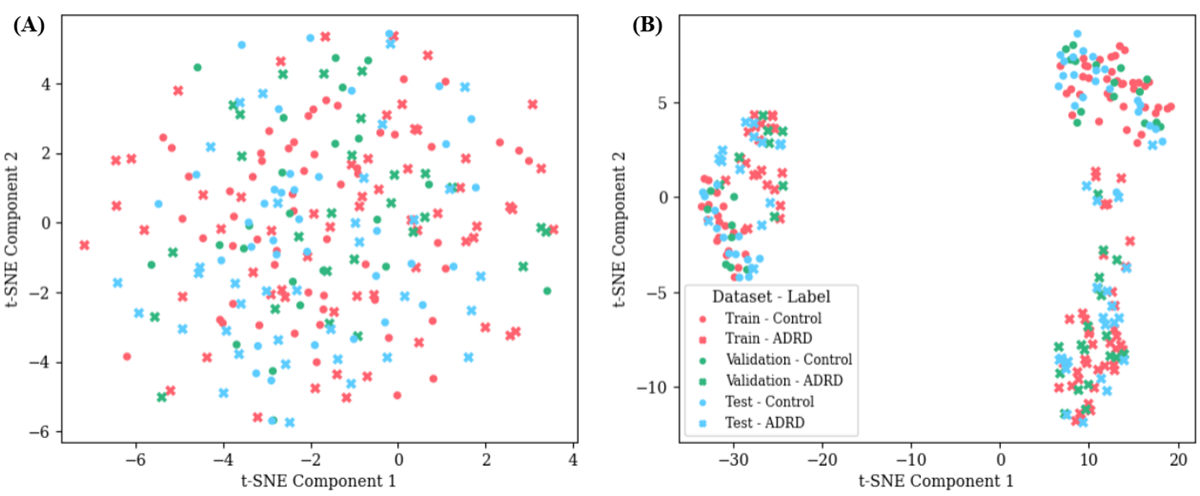
*t*-SNE visualization of linguistic and demographic feature spaces across dataset splits. (A) 2D *t*-SNE projection of word-level transcript embeddings. Points are color-coded by dataset split (train, validation, and test) and diagnosis (control vs ADRD). The extensive overlap indicates that all partitions occupy a comparable linguistic feature space, minimizing risk of distribution shift. (B) *t*-SNE projection of participant-level metadata vectors combining age, Mini-Mental State Examination, gender, recording duration, and word count. Three natural clusters reflect shared acoustic-demographic profiles, yet samples from every split and label are intermixed within each cluster, confirming balanced coverage of nonlinguistic characteristics across partitions. ADRD: Alzheimer disease and related dementia; *t*-SNE: *t*-distributed stochastic neighbor embedding.

To examine distributional similarity across partitions, we applied *t*-distributed stochastic neighbor embedding to word-level embeddings from the transcripts (Figure 2A) and to vectorized demographics (age, MMSE, gender, recording length, and word count (Figure 2B), providing insight into overlap among training, validation, and test sets.

### Text-Only LLMs Used in This Study

We evaluated 9 LLMs spanning diverse model sizes and training objectives. *GPT-4o* (text-only) served as a benchmark, representing a proprietary high-capacity model with advanced language understanding. *LLaMA 3.2 3B Instruct* [32] (LLaMA 3B), the smallest model, tested whether lightweight architectures can capture linguistic cues of cognitive impairment. *LLaMA 3.1 8B Instruct* (LLaMA 8B), a mid-sized model, was selected for its balance between efficiency and capacity to detect class-specific patterns. *MedAlpaca 7B* [34], fine-tuned on biomedical text, examined whether domain-specific pretraining enhances sensitivity to clinical language. *Ministral 8B* [33], optimized for efficient inference and strong text representation, evaluated the performance of general-purpose mid-sized models. *LLaMA 3.3 70B Instruct* (LLaMA 70B) and *LLaMA 3.1 405B Instruct* (LLaMA 405B), large and ultralarge open-weight models, tested the impact of scale on capturing linguistic signals. *Gemini 2.0 Flash* [37], a commercial model optimized for low-latency inference and embedded reasoning, was included for its potential to detect cognitive impairment-related cues. *DeepSeek-R1* [35], trained on diverse multilingual data, assessed whether alternative training paradigms generalize across speaker populations.

### LLM Adaptation Strategies for Cognitive Impairment Detection

#### Component 1: ICL with Demonstration Selection

ICL prompts were composed of four elements: an Instruction (IN), a set of demonstrations (DM), the test input ($T_{s}$), and the corresponding output label ($L_{s}$). The model estimates the conditional probability:


$$P\left(L_{s}\right|T_{s},\;IN,\;DM)$$

where each demonstration $DM_{k}=\left(Transcription_{k},Label_{k}\right)$ and $Label_{k}\in \{CN=0,CI=1\}$.

We began with a zero-shot baseline (ie, DM=∅), followed by few-shot experiments with N={2,4,6,8,10,12} demonstrations. All prompts were standardized in structure and length across models to control for prompt-induced variance. The prompt is presented in Multimedia Appendix 1.

To examine how the type of demonstrations influences performance, we evaluated four selection strategies: (1) most similar, (2) least similar, (3) average similarity to class prototypes, and (4) random. Each strategy selected N/2 demonstrations from each class (cognitive impaired and CN) to maintain balance.

Let $S_{i}^{C}$ denote the *i*th transcript from class C∈{CI,CN}, and let $E\left(S_{i}^{C}\right)\in R^{d}$ represent its semantic embedding computed using the Beijing Academy of Artificial Intelligence General Embedding transformer model [41]. For each test input $T_{s}$, we computed its embedding $E(T_{s})$, and calculated cosine similarity with all candidate demonstrations (from the separated training dataset):


$$Score\left(S_{i}^{C}\right)=\cos\left(E_{ref},E\left(S_{i}^{C}\right)\right)$$

where the reference embedding $E_{ref}$ was defined differently for each strategy:

- Most similar: $E_{ref}=E(T_{s})$. Select the top N/2 samples per class with highest cosine similarity to the test input.

- Least similar: $E_{ref}=E\left(T_{s}\right)$. Select the bottom N/2 samples per class with the lowest similarity to the test input.
- Average similar: $E_{ref}={\bar{E}}^{C}$, where ${\bar{E}}^{C}=\frac{1}{N^{C}}\sum_{k=1}^{N_{c}}E\left(S_{k}^{C}\right)$. Select the N/2 samples per class most similar to their class centroid, average of embeddings in each class.
- Random: Ignore similarity score and sample N/2 transcriptions per class uniformly at random.

Each strategy reflects a different hypothesis about which demonstrations best support generalization and reasoning:

- Most similar: examples provide contextual alignment, enhancing sensitivity to subtle cues.
- Least similar: examples increase linguistic variability, aiding generalization.
- Average similarity: samples serve as class prototypes, anchoring class distinctions.
- Random: serves as a baseline for assessing the general value of demonstrations.

We computed *F*_1_-scores for the cognitive impaired class on the validation set across all shot counts (n=2-12). The optimal n for each strategy was selected on the validation set and used for final evaluation on the held-out test set.

#### Component 2: Impact of Reasoning-Based Methods on Small LLMs

##### Overview

**Figure 3.**
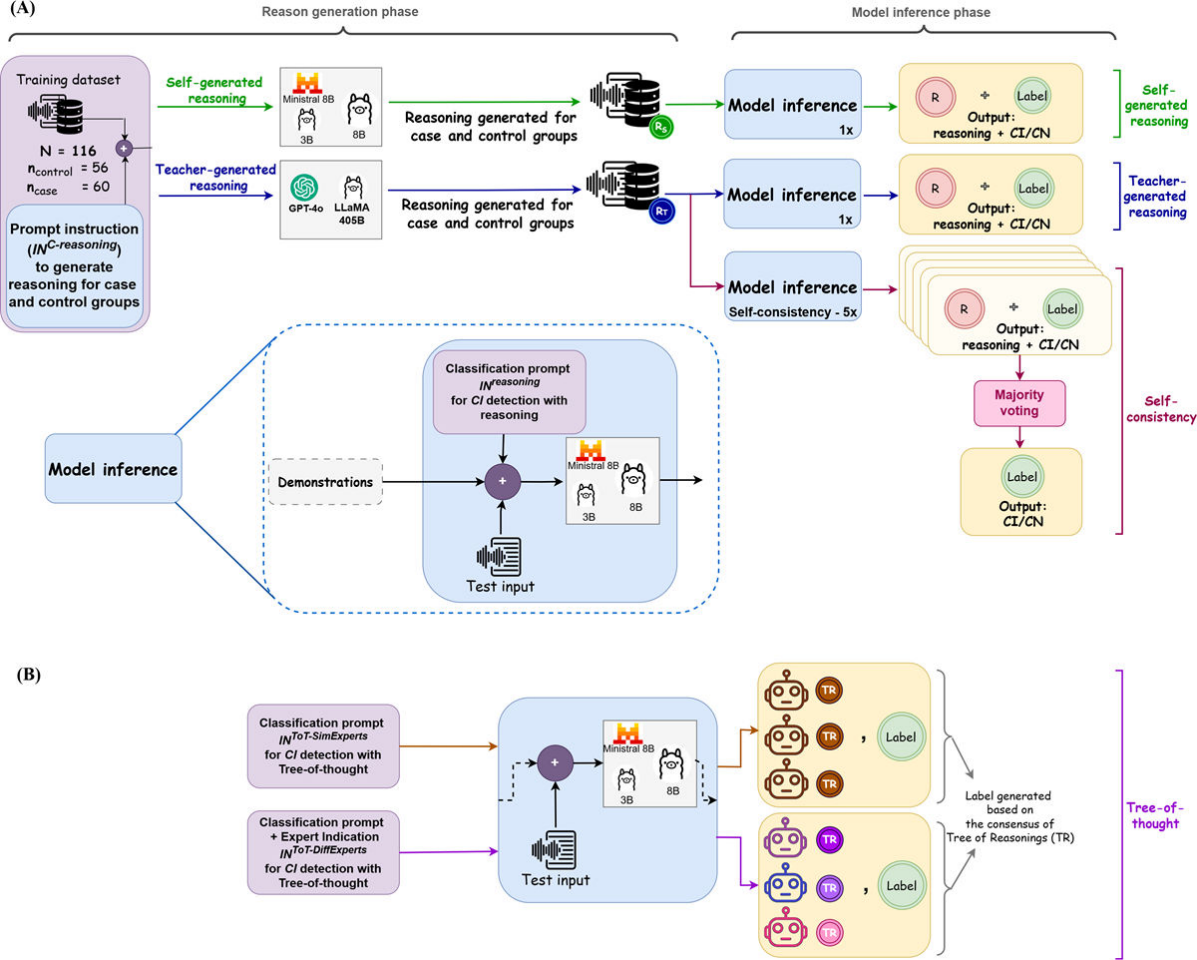
Overview of reasoning pipeline. (A) The methods include self-generated reasoning, teacher-generated reasoning, and self-consistency, where reasoning-augmented demonstrations are used for cognitive impaired or CN classification, and self-consistency aggregates multiple runs via majority voting. (B) Tree-of-thought pipeline, where the model is prompted to act as 3 experts, either unspecified or domain-specific, generate tree-of-reasoning (TR*)* and determine the final label by consensus. CI: cognitive impaired; CN: cognitive normal; ToT: tree-of-thought.

To assess whether explicit reasoning enhances classification accuracy in cognitive impairment detection, we evaluated three reasoning-based prompting strategies across three resource-efficient LLMs: LLaMA 3B, LLaMA 8B, and Ministral 8B Instruct (Figure 3). To support these smaller models, we incorporated rationales generated either by the models themselves (self-generated) or by larger teacher models (GPT-4o and LLaMA 405B).

##### Reasoning-Augmented In-Context Learning (Reasoning-ICL)

Reasoning-ICL augments each demonstration with an explanatory rationale alongside the input transcription and label, enabling the model to better associate linguistic features with cognitive status. Rationales were sourced from (1) self-generated explanations by the target model, and (2) teacher-generated rationales from a larger LLM (eg, GPT-4o or LLaMA 405B). For each combination, we computed the *F*_1_-score for the cognitive impaired class on the validation set and selected the best-performing shot count. The final performance was then evaluated on the held-out test set using this optimal configuration. See Multimedia Appendix 2 for prompt design.

Formally, for each training transcription $S_{k}^{C}$, a rationale ${Reason}_{k}^{x}$ was generated, where x∈{self,teacher} indicates the source of the explanation. Each demonstration is a triplet:

$DM_{k}^{reason}=(S_{k}^{C},Reason_{k}^{x},Label_{k})$

where $Label_{k}\in (CI,CN)$

At inference, the target LLM received the test input $T_{s}$, a reasoning-specific instruction ${IN}_{reason}$, and a set of augmented demonstrations ${DM}_{K}^{reason}$ (chosen by the “Average” demonstration selection strategy introduced in component 1), then jointly generated both a rationale and classification label:


$$P\left(Reason_{s},L_{s}|T_{s},IN_{reason},DM_{K}^{reason}\right)$$

This framework enabled us to test whether adding structured rationales, generated by either the model itself or a more capable teacher model, improves the model’s ability to detect cognitive impairment.

##### Self-Consistency With Teacher-Generated Reasoning

To assess whether reasoning-augmented ICL could be further improved by reducing variability in model outputs, we implemented the self-consistency [42] method, which aggregates predictions across multiple independently sampled inference runs using a fixed prompt. We restricted self-consistency to teacher-generated rationales, as results from *Results—Component 2: Self-consistency with teacher-generated reasoning* (see Results) showed that teacher-based reasoning using rationales from LLaMA 405B consistently outperformed self-generated rationales, GPT-4o rationales, and nonreasoning prompts across most shot counts.

For each shot count 2 to 12, we used demonstrations augmented with teacher-generated rationales ${DM}_{K}^{reason}$. Each test input $T_{s}$ was processed 5 times using the same instruction (${IN}_{reason}$ ) and the same set of demonstrations, under two temperature settings: 0.0 for deterministic decoding and 0.5 to introduce controlled randomness. Each run produced a pair: a generated rationale and a corresponding label ($Reason_{s}$, $L_{s}$).


$$P^{i}(Reason_{S},L_{S}|T_{S},IN_{reason},DM_{K}^{reason}),i=1,\ldots ,5$$

The final predicted label $\overset{⌢}{L_{s}}$ was computed by majority vote over the 5 predicted labels $L_{s}^{\left(1\right)}$, …, $L_{s}^{\left(5\right)}$ using the below formula.


$$L_{s}=\underset{l\in (\mathrm{ADRD},\mathrm{Healthy})}{\mathrm{argmax}}\sum_{i=1}^{5}1\{L_{s}^{\left(i\right)}=l]$$

##### Tree-of-Thought Reasoning

To evaluate a structured, multistep reasoning approach beyond self-consistency, we implemented the tree-of-thought (ToT) [43] prompting framework. This method guides the model to break down decisions into intermediate steps, allowing it to generate and evaluate multiple reasoning paths before producing a final classification. By reasoning step-by-step, the model can retain, revise, or discard partial thoughts, potentially improving coherence and robustness.

We adopted a zero-shot setup to assess ToT’s effectiveness independently of in-context demonstrations. For each test input, the model was prompted to reason from the perspective of 3 simulated experts, each generating a short sequence of reasoning steps. We tested the following 2 prompt formats:

1. *Unspecified experts:* experts introduced with “Imagine three different experts are analyzing a speech transcript.”
2. *Domain-specific experts: *experts identified as a language and cognition specialist, a neurocognitive researcher, and a speech-language pathologist.

Each expert generated up to 2 sequential reasoning steps before providing a final classification. This corresponds to a tree with depth 2 and breadth 3. We capped the depth at 2 steps, as additional steps often led to repetitive or uninformative outputs.

This setup enabled evaluation of ToT as a standalone reasoning strategy without demonstrations, while maintaining consistent prompt structure and model size across methods. Full prompt templates are provided in Multimedia Appendix 3.

### Component 3: Fine-Tuning for Binary Classification

To assess whether task-specific adaptation improves model performance, we fine-tuned a subset of LLMs to classify transcripts as either cognitively impaired or CN. We implemented 2 approaches to fine-tuning.

#### Token-Level Supervised Fine-Tuning

In this approach, classification was framed as a next-token prediction task. Each transcript was paired with a task-specific prompt (Multimedia Appendix 4), and the model was trained to generate the target label token, “AD” (cognitive impaired) or “Healthy” (CN). The objective was token-level cross-entropy loss over the model’s vocabulary, with the correct label token as the target.

Fine-tuning was applied to open-weight models (LLaMA 3B, LLaMA 8B, LLaMA 70B, MedAlpaca 7B, and Ministral) using Low-Rank Adaptation (LoRA) [44] for parameter-efficient optimization. We used LoRA to constrain fine-tuning to a low-rank update of a small subset of parameters, reducing effective model capacity and thereby lowering the risk of memorization and overfitting in this limited-data setting. We performed a grid search over LoRA rank (32, 64, and 128), dropout (0.00, 0.05, and 0.10), learning rate (2e-4), and batch size (4, 8, and 16), with training epochs from 1 to 13. The best configuration for each model was selected based on the *F*_1_-score for the cognitive impaired class on the validation set (Multimedia Appendix 5). To quantify training variability due to random initialization and data order, we repeated fine-tuning across 5 distinct random seeds (controlling both data shuffling and adapter initialization) and report the mean *F*_1_-score with 95% CIs.

For commercial models (GPT-4o and Gemini-2.0), we used application programming interface (API)-level fine-tuning options, including learning rate multipliers, training epochs, and adapter or batch size where applicable. Hyperparameter choices were guided by API documentation and prior work. As with open-weight models, final settings were selected based on the *F*_1_-score for the cognitive impaired class on the validation set.

At inference, temperature was fixed at 0.0 for deterministic decoding. Where available, we also extracted class probabilities to compute threshold-independent metrics, specifically the area under the receiver operating characteristic curve. These probabilities were derived from SoftMax-normalized logits assigned to the “AD” and “Healthy” tokens in the output layer (see Multimedia Appendix 6 for more details).

#### Classification Head Fine-Tuning

In this approach, we reframed the classification task by appending a lightweight classification head to the final hidden state of the LLM. The head consisted of 3 fully connected layers (output size: vocabulary dimension → 512 → 256 → 2), following the standard architecture used in Hugging Face implementations [45]. It was trained using binary cross-entropy loss to directly map hidden representations to class probabilities.

Unlike the token-level method, this approach decouples classification from language generation, allowing the model to learn class-specific features from its internal states rather than relying on token prediction. This method was applied only to open-weight models, where hidden representations are accessible.

Training inputs, prompts, and hyperparameter tuning followed the same procedures as in the first approach (MultimediaAppendices 4  5). During inference on the held-out test set, the classification head generated logits for each class, which were then converted into labels for evaluation.

### Component 4: Evaluating Multimodal LLMs as Classifier

To evaluate multimodal LLMs for cognitive impairment classification, we tested 3 state-of-the-art models using paired audio and transcripts. All models were prompted to process both modalities and output the patient’s cognitive status. Multimedia Appendix 7 includes details of this prompt.

- *GPT-4o mini* [36]: OpenAI’s closed-weight model supporting text and audio inputs. Due to limited access, we performed zero-shot inference using the API with temperature set to 0.
- *Qwen 2.5 Omni *[46]: Evaluated using two strategies:
  - Zero-shot*:* Run with Hugging Face’s recommended parameters (eg, temperature=1.0, top-k=50, top-p=1.0).
  - Fine-tuning: Performed using LLaMA Factory on training-set audio-transcript pairs. LoRA was used for efficient adaptation with recommended hyperparameters [46] (see Multimedia Appendix 8 for details).
- *Phi-4 Multimodal *[47]: Microsoft’s multimodal successor to the Phi series.
  - Zero-shot*:* Run with Hugging Face’s recommended parameters (eg, temperature=1.0, top-k=50, top-p=1.0) using the same instruction prompt as Qwen and GPT-4o.
  - Fine-tuning*:* Conducted via Hugging Face by a grid search over gradient accumulation steps, number of epochs, and audio length and with recommended LoRA-based settings [47] (see Multimedia Appendix 8 for details and ablation studies).

For both Qwen and Phi-4, the number of epochs was selected based on the validation of the *F*_1_-score for the cognitive impaired class.

### Error Analysis

#### Overview

We selected fine-tuned GPT-4o and LLaMA 8B for error analysis because they showed consistent high performance across adaptation strategies and represent two practical deployment settings. LLaMA 8B represents a strong open-weight, parameter-efficient model that can be deployed locally in low-resource or privacy-sensitive clinical settings, whereas GPT-4o represents a high-capacity commercial model that operates through an external service. By including one open-weight model and one commercial model, we aimed to provide error insights that are relevant to different deployment settings.

#### Qualitative Analysis

Two team members with expertise in audio analysis independently reviewed all misclassified cases in the held-out test set, including false positives (FP; CN predicted as impaired) and false negatives (FN; cognitive impaired predicted as normal). For each case, reviewers listened to the raw audio and examined the corresponding AWS transcribe output. Each misclassification was annotated for the presence of (1) noise in the audio and (2) missing or partial transcription. We adopted this manual review approach because these error metrics cannot be reliably assessed using automated metrics alone (eg, signal-to-noise ratio-based measures) and due to the lack of gold-standard transcripts.

#### Quantitative Analysis

We computed 25 text-derived metrics across four domains—lexical richness (11), syntactic complexity (7), disfluency or repetition (2), and semantic coherence (5) (Multimedia Appendix 9)—for all test samples. These domains were included because initial analysis showed that the automatic measures were reliable and captured several error patterns observed during human review. Distributions were compared using a 2-sided Mann-Whitney *U* test [48] for true positive (TP) versus FN within the cognitive impaired group and true negative (TN) versus FP within the CN group. A *P* value less than .10 was used to flag potential differences.

### Generalization Beyond ADReSSo: External Validation on Delaware Dataset

We evaluated the performance of three components on the DementiaBank Delaware dataset [49], which includes 3 picture-description tasks (Cookie Theft, Cat Rescue, and Rockwell), a Cinderella story recall, and a procedural discourse task. The dataset consists of 205 English-speaking participants (n=99 with MCI, and n=106 CN).

We performed a 60%-20%‐20% participant-level split for training (n=124), validation (n=40), and testing (n=41), ensuring that recordings from each participant appeared in only one split. To calculate the *F*_1_-score, we aggregated the predictions for each participant across all tasks, applying majority voting to the predicted labels and comparing them to the ground-truth labels.

On this dataset, we evaluated adaptation strategies described in component 1‐3:

- Component 1: ICL with demonstration selection—We focused on LLaMA 8B and GPT-4o because they showed high and stable performance across demonstration selection strategies. Specifically, we presented the results of the *Most Similar* demonstration selection strategy because it generally outperformed other strategies.
- Component 2: Impact of reasoning-based methods on small LLMs—Specifically, we used Reasoning-Augmented ICL for LLaMA 8B using rationales generated by LLaMA 405B, which showed the highest performance in our ADReSSo-related experiments.
- Component 3: Fine-tuning for binary classification—Similar to ICL, we evaluated LLaMA 8B and GPT-4o, which outperformed other open-weight and commercial LLMs in token-level supervised fine-tuning. Both LLMs were trained on the Delaware training set with hyperparameters selected on the validation set, following the hyperparameter selection procedure described for ADReSSo. We repeated fine-tuning across five distinct random seeds and reported the mean performance with 95% CIs.

### Ethical Considerations

The data used in this study were obtained from the Pitt Corpus and Delaware Corpus in the DementiaBank database, a publicly available resource hosted by TalkBank. Pitt’s original data collection was approved by the Institutional Review Board of the University of Pittsburgh. Delaware’s collection was supported by the National Institute of Aging of the National Institutes of Health under award number RF1AG083823. As this study involved secondary analysis of deidentified data, no additional Institutional Review Board approval was required. Informed consent was obtained from participants in the original studies that contributed data to the DementiaBank database.

## Results

Throughout this section, *F*_1_-scores refer to the cognitive impaired class unless otherwise specified.

### Component 1: ICL with Demonstration Selection

**Figure 4.**
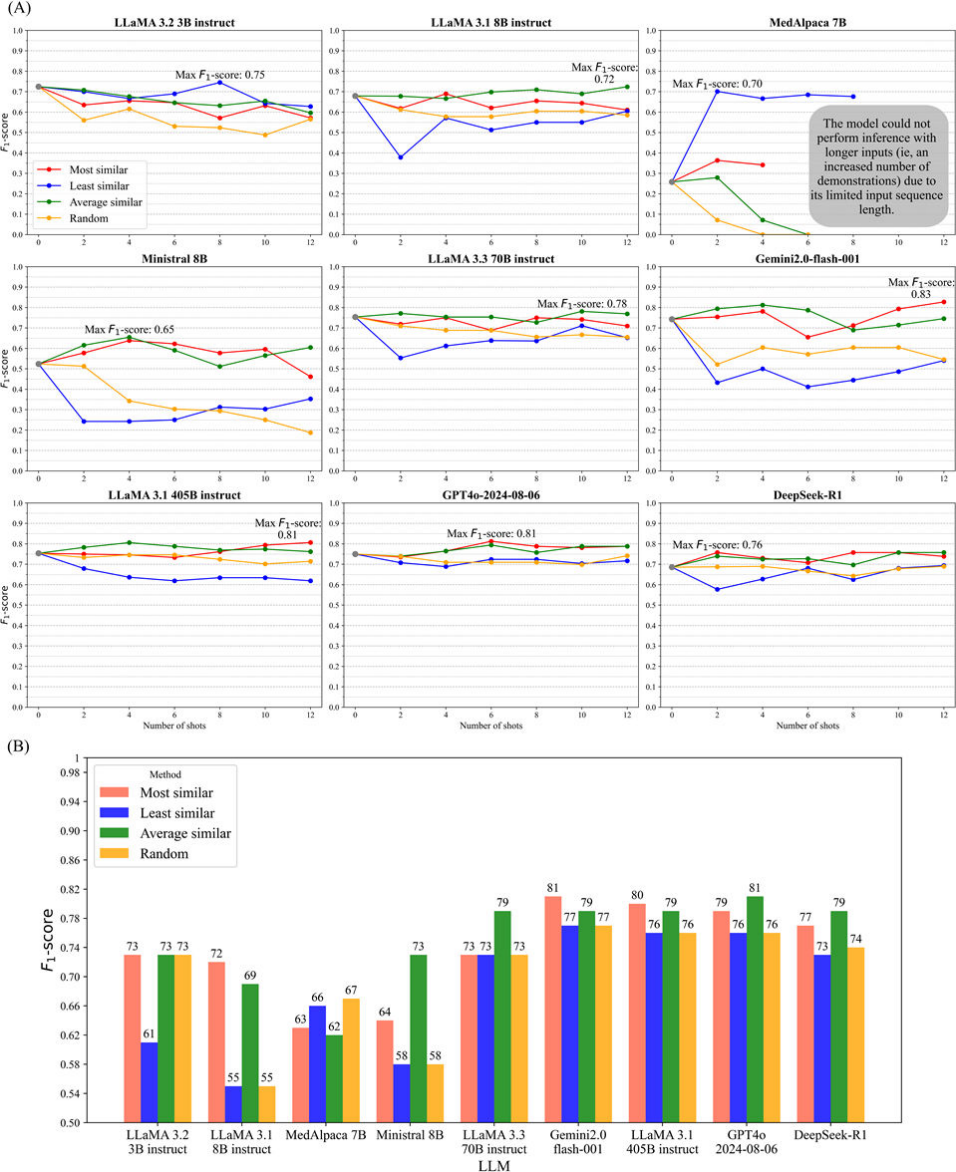
Impact of demonstration selection strategies on in-context learning performance across LLMs. (A) Results for validation: *F*_1_-scores for 2-12 demonstrations show average similarity often outperforming other methods, with larger models plateauing after approximately 6 shots and smaller models showing greater sensitivity to selection quality. (B) Results for test: Using optimal shot counts from (A), average similarity achieves the highest scores for most models, while most similar leads in a few cases. Numbers above bars indicate *F*_1_-scores ×100*.* LLM: large language model.

Figure 4A presents validation *F*_1_-scores for each LLM using 2 to 12 in-context demonstrations across 4 selection strategies. Demonstrations selected by *average similarity* to class centroids achieved the highest or joint-highest *F*_1_-scores in 5 models and ranked second in 3 others. The *most similar* strategy generally produced the next-best performance, with notable results for GPT-4o and Gemini-2.0. *Least similar* examples yielded the lowest scores overall, except for MedAlpaca-7B and LLaMA 3B. *Random* selection showed minimal improvement over zero-shot, suggesting limited benefit from unstructured examples. In larger models, performance gains plateaued after 6 demonstrations, indicating reduced sensitivity to demonstration quality, whereas smaller models remained more influenced by selection strategy.

Figure 4B shows corresponding results on the test set. *Average similarity* achieved the highest *F*_1_-scores in 5 models, including LLaMA 3B (0.73), Ministral 8B (0.73), LLaMA 70B (0.79), GPT-4o (0.81), and DeepSeek-R1 (0.79). *Most similar* was optimal for LLaMA 8B (0.72), LLaMA 405B (0.80), and Gemini-2.0 (0.81). *Least similar* continued to underperform, while MedAlpaca-7B again performed best with random samples (*F*_1_=0.67). These results highlighted the importance of selecting representative, class-central demonstrations to enhance generalization in ICL.

### Component 2: Impact of Reasoning-Based Methods on Small LLMs

#### Reasoning-Augmented In-Context Learning (Reasoning-ICL)

**Figure 5.**
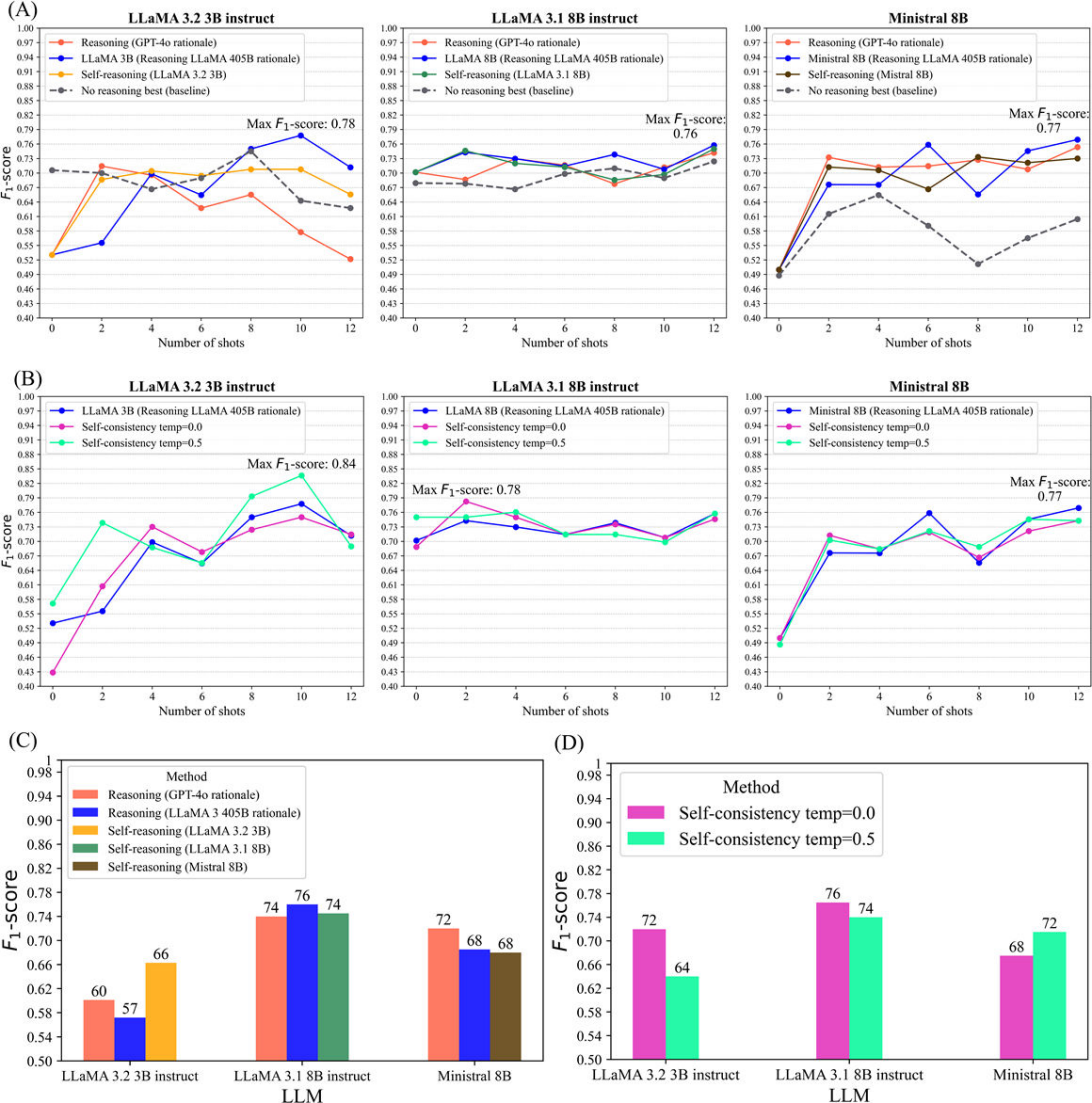
Reasoning-augmented in-context learning and self-consistency performance on small LLMs. (A) Reasoning-augmented in-context learning (validation): Adding rationales, especially those generated by LLaMA 405B, improved *F*_1_-scores over the no-reasoning baseline, with the largest gains in LLaMA 3B and Ministral 8B. (B) Self-consistency (validation): Majority voting over multiple outputs with LLaMA 405B–generated rationales showed minor changes, with temperature adjustments having limited benefit. (C) Reasoning-augmented in-context learning (test): Performance trends differed from validation; unlike validation, best scores varied by model and rationale source. (D) Self-consistency (test): Majority voting slightly improved stability and accuracy for some LLaMA 3B and not for larger models. Numbers above bars indicate *F*_1_-scores × 100*.* LLM: large language model.

Validation results (Figure 5A) indicated that adding rationales improved *F*_1_-scores across all 3 small LLMs compared with the no-reasoning baseline. Rationales generated by LLaMA 405B yielded the largest gains. With 10 demonstrations, LLaMA 3B achieved an *F*_1_-score of 0.78 (vs 0.64 baseline), while with 12 shots, Ministral 8B reached 0.77 (vs 0.61), and LLaMA 8B reached 0.76 (vs 0.72). Rationales from GPT-4o consistently outperformed self-generated rationales but were generally below LLaMA 405B across most shot counts.

Test-set results (Figure 5C) were less aligned with validation trends. GPT-4o rationales produced the highest *F*_1_-score for Ministral 8B (0.72), while LLaMA 405B rationales yielded the best result for LLaMA 8B (0.78). Notably, LLaMA 3B performed best (0.66) with self-generated rationales. These discrepancies indicate that validation-set trends may not reliably reflect a model’s generalization behavior, and that performance improvements from specific rationale sources or shot counts should be interpreted with caution.

#### Self-Consistency With Teacher-Generated Reasoning

Using LLaMA 405B-generated rationales, we sampled multiple outputs per input and aggregated predictions by majority vote (Figure 5B). Even with temperature=0, repeated inferences produced slight variations, reflecting the inherent stochasticity of LLMs. Aggregating predictions via majority voting left LLaMA 8B performance unchanged, but reduced *F*_1_-scores from 0.78 to 0.75 for LLaMA 3B and from 0.77 to 0.74 for Ministral 8B. Using a moderate temperature (0.5) increased output variation without improving performance.

On the test set (Figure 5D), though self-consistency improved model performance, it did not preserve the validation trend. Majority voting at a temperature of 0.0 increased the *F*_1_-score by 0.005 and 0.05 for LLaMA 3B, resulting in a score of 0.72, and LLaMA 8B, resulting in a score of 0.76, with respect to the performance of LLaMA 405 rationales, and lowered by 0.01 for Ministral 8B, reaching 0.67. Results at a temperature of 0.5 were lower for the LLaMA family and only improved Ministral 8B’s performance to 0.71. These findings highlight self-consistency as an effective strategy for mitigating prediction instability, an intrinsic property of LLMs, even under deterministic decoding settings.

#### ToT Reasoning

In zero-shot classification, the 3 evaluated models—LLaMA 3B, LLaMA 8B, and Ministral 8B—achieved baseline *F*_1_-scores of 0.73, 0.55, and 0.57, respectively. Applying ToT prompting with unspecified expert roles altered performance to 0.59 (−0.10), 0.63 (+0.11), and 0.66 (+0.09) for LLaMA 3B, LLaMA 8B, and Ministral 8B, respectively. When domain-relevant expert roles were incorporated, *F*_1_-scores increased to 0.68 (+0.09 vs nonexpert), 0.71 (+0.05), and 0.69 (+0.03), respectively. Compared with zero-shot, expert-role ToT produced notable gains for LLaMA 8B (+0.16) and Ministral 8B (+0.12), but remained below baseline for LLaMA 3B (−0.05).

These findings indicate that expert-grounded prompting can enhance large model performance in cognitive impairment classification, whereas the smaller model, despite benefiting most from expert-role ToT relative to its nonexpert counterpart, may lack the capacity for sustained multi-step reasoning.

### Component 3: Fine-Tuning for Binary Classification

#### Token-Level Supervised Fine-Tuning

**Figure 6.**
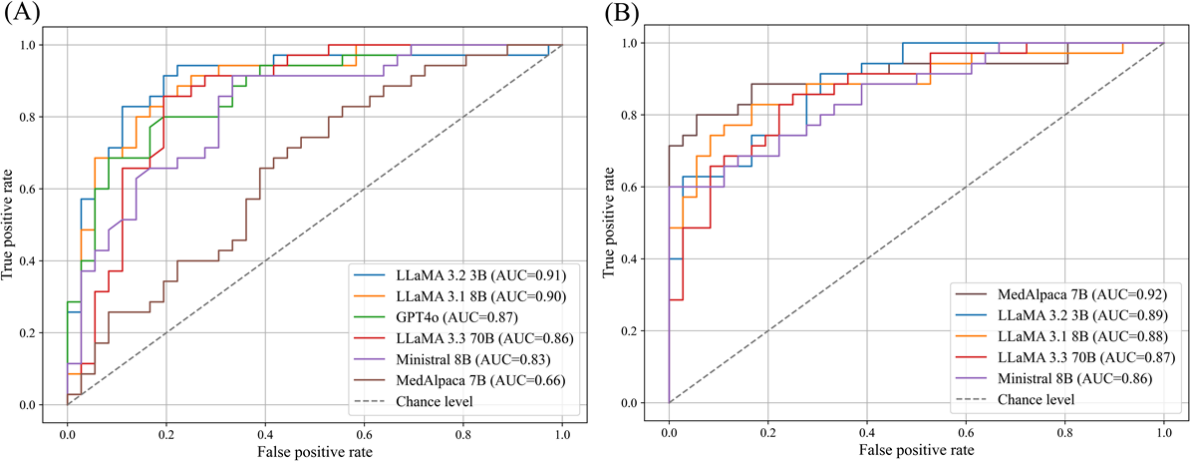
*C*omparison of token-level and classification-head fine-tuning for binary classification on the test set. (A) Token-level fine-tuning shows strong AUC performance for most models, with LLaMA 3B and LLaMA 8B leading and MedAlpaca 7B lagging. (B) Classification-head fine-tuning markedly boosts MedAlpaca 7B but reduces performance for models already strong under token-level training. AUC: area under the curve.

Figure 6 compares token-level supervised fine-tuning and classification-head fine-tuning across 6 models, reporting area under the curve [AUC] for the best-performing configuration of each model. Under token-level supervision (Figure 6A), LLaMA 3B and LLaMA 8B achieved the highest AUCs (0.91 and 0.90) and corresponding *F*_1_-scores of 0.83, 95% CI 0.01 and 0.81, 95% CI 0.01, respectively. These were followed by GPT-4o (AUC=0.87, *F*_1_=0.79, 95% CI 0.01), LLaMA 70B (AUC=0.86, *F*_1_=0.82, 95% CI 0.02), Ministral 8B (AUC=0.83, *F*_1_=0.77, 95% CI 0.01), and MedAlpaca 7B (AUC=0.66, *F*_1_=0.06, 95% CI 0.02). Performance patterns indicate that smaller and mid-sized models achieved strong class separability, whereas MedAlpaca 7B underperformed, likely due to tokenization-related mismatches with the task data.

To assess potential overfitting due to validation-based hyperparameter selection, we additionally report validation and test *F*_1_-scores for all token-level fine-tuned models in Table 3 in Multimedia Appendix 5. Across models, validation *F*_1_-scores are high and closely aligned with the corresponding test *F*_1_-scores, indicating that hyperparameter selection using the validation set did not lead to substantial overfitting, as performance generalized consistently to the held-out test set.

#### Classification Head Fine-Tuning

In contrast, classification-head fine-tuning (Figure 6B) substantially improved MedAlpaca 7B (AUC=0.92, *F*_1_=0.81, 95% CI 0.04, +0.75 improvement), while LLaMA 3B and LLaMA 8B declined to AUC values of 0.89 and 0.88 (*F*_1_=0.75, 95% CI 0.03 and 0.80, 95% CI 0.01, respectively). Although Ministral 8B and LLaMA 70B increased the AUC to 0.86 and 0.87, their *F*_1_-scores dropped to 0.74, 95% CI 0.02 and 0.79, 95% CI 0.02, respectively. These results suggest that classification-head fine-tuning can markedly benefit models that perform poorly with token-level supervision, while models already performing well under token-level training may not gain, and can even lose, performance when switching to a classification-head approach.

### Component 4: Evaluating Multimodal LLMs as Classifier

The following findings have been observed:

- *GPT-4o Mini:* In the zero-shot setting, GPT-4o Mini achieved a high *F*_1_-score for cognitive impaired cases (0.70) but only 0.29 for CN cases, indicating substantial bias toward predicting impairment. Fine-tuning was not performed due to OpenAI’s access limitations.
- *Qwen 2.5-Omni:* Zero-shot performance yielded an *F*_1_-score of 0.70 for CN cases and 0.54 for cognitive impaired cases, reflecting a reverse bias toward predicting CN. Fine-tuning did not improve performance and failed to address this imbalance.
- *Phi-4 Multimodal:* Zero-shot performance was balanced, with *F*_1_-scores of 0.53 for cognitive impaired and 0.51 for CN cases. Fine-tuning led to substantial gains, reaching 0.80 for cognitive impaired and 0.75 for CN cases, the highest overall performance and largest improvement among all models.

These findings indicate that while GPT-4o Mini and Qwen 2.5-Omni performed reasonably in zero-shot mode, both exhibited strong class biases and limited benefit from fine-tuning. In contrast, Phi-4 Multimodal maintained balanced zero-shot performance and responded strongly to fine-tuning, underscoring the importance of task-specific training for robust CN classification.

### Error Analysis

#### Misclassification Overview

**Figure 7.**
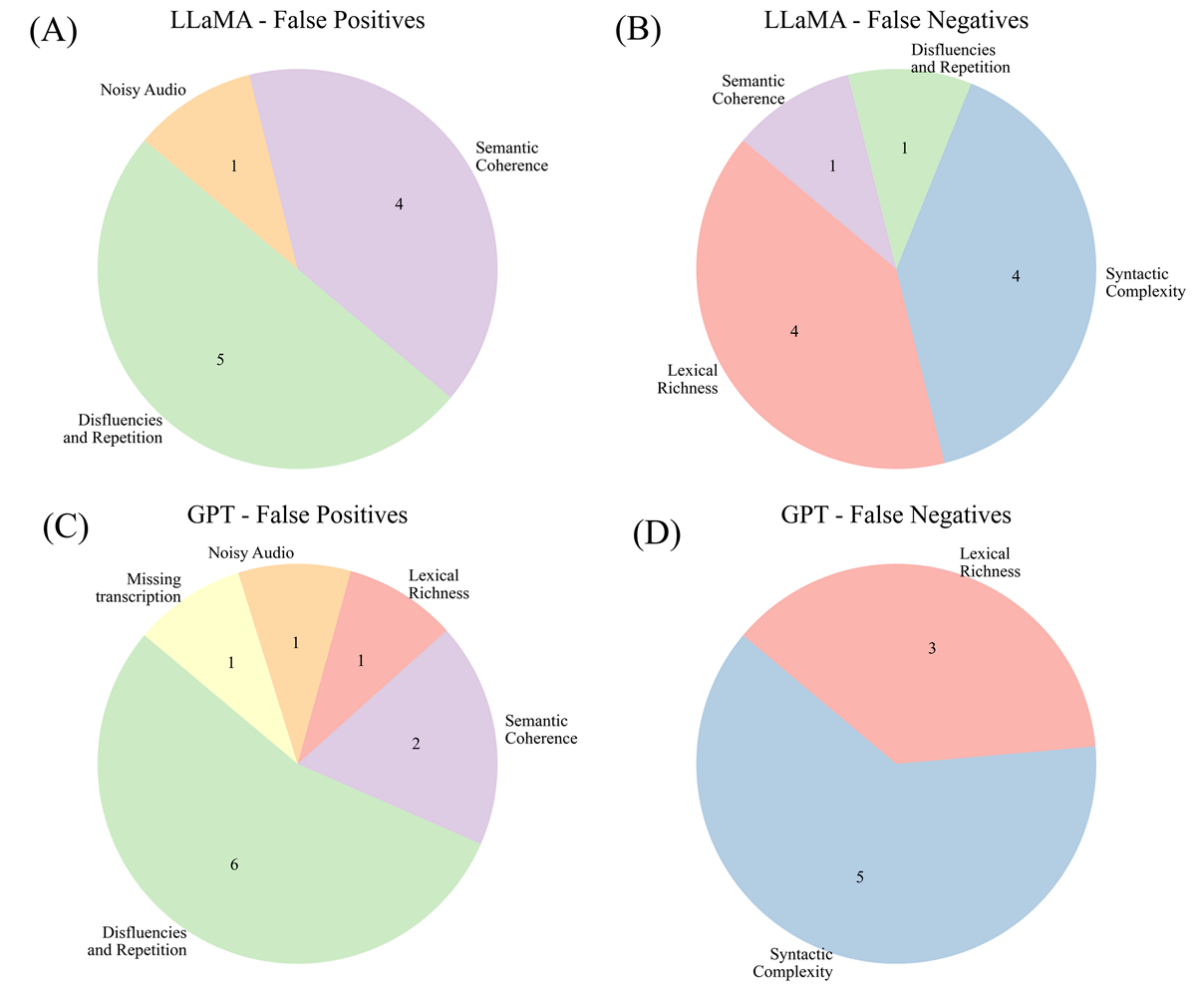
Distribution of linguistic and technical issues contributing to model misclassifications on the test set. (A) LLaMA 8B false positives were primarily due to disfluencies or repetition and semantic coherence. (B) LLaMA 8B false negatives were mainly linked to lexical richness and syntactic complexity. (C) GPT-4o false positives were dominated by disfluencies/repetition, with smaller contributions from semantic coherence and transcription-related issues. (D) GPT-4o false negatives were largely associated with syntactic complexity and lexical richness.

On the held-out test set (n=71), LLaMA 8B produced 6 FNs (TN=30) and 7 FNs (TP=28); GPT-4o produced 8 FPs (TN=28) and 7 FNs (TP=28). Three FPs and 4 FNs overlapped across models. Figure 7 summarizes error metrics for LLaMA 8B and GPT-4o.

#### Qualitative Analysis

Two problematic cases were excluded: 1 involved noisy audio with overlapping speech that produced an unrelated transcript and was misclassified by both models as an FP; the other had a missing transcription despite a high-quality audio file and was misclassified by GPT-4o as an FP. The remaining samples were used for quantitative analysis.

#### Quantitative Analysis

Mann-Whitney *U* tests (*P* value <.10) showed significant feature differences between correct and incorrect predictions. For GPT-4o, disfluencies or repetition differed for TP versus FN and TN versus FP, and semantic coherence differed for TN versus FP. For LLaMA 8B, syntactic complexity and semantic coherence differed for TP versus FN; lexical richness, semantic coherence, and syntactic complexity differed for TN versus FP. Together, these results suggest that misclassifications arise when a sample’s linguistic profile resembles that of the opposite class.

A limitation of this analysis is that all automatic speech transcription systems are prone to word insertions, repetitions, and truncations, which may have contributed to some of the observed errors and are reflected in our error categorization.

### External Generalizability Evaluation: DementiaBank Delaware Dataset

We evaluated components 1-3 as follows:

- Component 1: ICL with demonstration selection—LLaMA 8B achieved an *F*_1_-score of 0.74 with 4 demonstrations for the Most Similar demonstration strategy, while GPT-4o performed better with only 2 demonstrations, reaching an *F*_1_-score of 0.80. Adding further demonstrations led to a decline in GPT-4o’s performance, suggesting that GPT-4o may be more sensitive to demonstration noise, where additional examples dilute task-relevant signals rather than providing incremental benefit.
- Component 2: Impact of reasoning-based methods on small LLMs—We next evaluated LLaMA 8B with reasoning-augmented ICL, using rationales generated by LLaMA 405B. This approach resulted in an *F*_1_-score of 0.75 with 10 shots on the Delaware dataset, suggesting that reasoning-based ICL can further enhance performance on smaller models by improving the quality of the input prompts.
- Component 3: Fine-tuning for binary classification—Finally, we fine-tuned both LLaMA 8B and GPT-4o on the Delaware dataset using similar prompts used with the AdReSSo dataset. Token-level fine-tuning led to an improvement for both models: LLaMA 8B reached an *F*_1_-score of 0.76, 95% CI 0.02, and GPT-4o reached an *F*_1_-score of 0.82, 95% CI 0.03. These results confirm the benefits of fine-tuning LLMs on task-specific data.

## Discussion

### Principal Results

This study presents the first comprehensive evaluation of multiple adaptation strategies, ICL, reasoning-augmented ICLs, self-consistency, ToT, and supervised fine-tuning across state-of-the-art open-weight and commercial LLMs for detecting early cognitive impairment from speech transcripts (ADReSSo subset of the Pitt Corpus). Fine-tuning yielded the strongest performance: LLaMA 3B, LLaMA 70B, and LLaMA 8B achieved *F*_1_-scores of 0.83, 95% CI 0.01; 0.82, 95% CI 0.02; and 0.81, 95% CI 0.01, respectively, outperforming GPT-4o (*F*_1_=0.79, 95% CI 0.01). These results show that small open-weight models, when adapted to domain-specific tasks, can match or exceed commercial models, offering practical advantages in scalability and deployment.

In the context of ICL, the demonstration selection strategy proved critical to performance. Demonstrations selected based on average similarity to class centroids, intended to reflect prototypical speech patterns of CN and impaired individuals, outperformed those based on most similar, least similar, or random selection. This effect was observed across both small and large models, with performance gains plateauing after six examples in larger models. These results highlight the importance of representative, class-central exemplars for guiding model generalization, especially in clinical tasks where linguistic variability may obscure diagnostic signals.

Teacher-generated rationales from LLaMA 405B or GPT-4o improved reasoning-augmented ICL for smaller models, increasing the *F*_1_-score of LLaMA 8B from 0.72 to 0.76. This suggests that teacher-generated reasoning can guide models toward better predictions, reducing adaptation costs by substituting for manually labeled examples. Self-consistency—aggregating predictions from repeated runs—boosted LLaMA 3B from 0.66 to 0.72 but offered limited benefit for larger models. These findings suggest that self-consistency mitigates prediction variability in smaller LLMs but is less impactful in models with more stable outputs.

We also observed discrepancies in the performance of some LLMs across the validation and test sets. For example, the average similar demonstration selection strategy yielded the highest *F*_1_-score on the validation set for LLaMA 8B, whereas the most similar demonstrations achieved the highest *F*_1_-score on the test set. Similarly, in the augmented-reasoning ICL component, LLMs achieved the highest *F*_1_-scores with rationales generated by different teacher models on the validation and test sets. These discrepancies were more pronounced in smaller LLMs, which tend to be less generalizable and more sensitive to input variations. As mentioned earlier, the validation set was drawn from the stratified ADReSSo development data, whereas the test set followed the official ADReSSo split and appears to contain more challenging cases with somewhat different linguistic profiles. Such differences in data distribution may account for the observed performance discrepancies in smaller models. Therefore, we recommend interpreting validation scores for smaller, non–fine-tuned LLMs with caution and adopting multiple adaptation strategies rather than relying on a single “best” strategy.

Token-level fine-tuning outperformed classification-head adaptation for most models. An exception was MedAlpaca-7B, which performed poorly in the token-based setup (*F*_1_=0.06, 95% CI 0.02 for cognitive impairment class), likely due to its difficulty generating the correct label token during inference. However, when trained with a classification head, its performance improved substantially (*F*_1_=0.81, 95% CI 0.04 for cognitive impairment class). Overall, these results suggest that the optimal fine-tuning formulation depends on how reliably a model can produce discrete label tokens. It is worth mentioning that although fine-tuning LLMs outperforms other adaptation strategies, it might reduce LLMs’ generalizability for data that lie outside the training data distribution. For example, LLMs fine-tuned on the Cookie Theft picture description task may not result in the best performance on other speech tasks such as story recall.

Hyperparameter choices for fine-tuning LLMs are dataset- and model-specific and should be re-evaluated when applying fine-tuning to new benchmarks or data from different clinical settings. In this study, we limited hyperparameter selection to a constrained, literature-informed grid (eg, LoRA or quantized low-rank adaptation rank, dropout, learning rate, batch size, and epochs) and selected configurations using a stratified validation subset drawn from the ADReSSo development set. We further evaluated robustness by repeating fine-tuning across 5 random seeds, reporting mean *F*_1_-score with 95% CIs, and examining validation-test consistency and ablations (Tables 3-5, Multimedia Appendix 5). The selected hyperparameter values are intended to serve as practical starting points for future work rather than universally optimal settings.

Overall, multimodal LLMs underperformed relative to text-only LLMs in our experiments. In zero-shot settings, GPT-4o Mini and Qwen 2.5-Omni exhibited pronounced class bias, favoring the cognitive impaired class in GPT-4o Mini and CN class in Qwen 2.5-Omni. Even after fine-tuning, Phi-4 Multimodal (*F*_1_=0.80 for cognitive impaired; *F*_1_=0.75 for CN) did not match the performance of the best text-only models. These findings suggest that the large, audio branches of current multimodal LLMs are difficult to adapt in small clinical datasets and may introduce variability that propagates errors into the joint audio-text representation rather than providing consistently complementary information. This limitation is likely driven by a combination of insufficient task-specific speech supervision during the process of training and the substantial data requirements for fine-tuning, rather than by a lack of informative acoustic cues in speech itself.

Consistent with this interpretation, prior work using smaller, speech-focused models such as Wav2Vec and mHuBERT has demonstrated that audio-based markers of cognitive impairment can be learned effectively on datasets of comparable scale. These models benefit from substantially fewer parameters and pretraining objectives explicitly tailored to speech, enabling more efficient adaptation to clinical speech tasks. Together, these results indicate that the observed underperformance of multimodal LLMs reflects current architectural and data-efficiency limitations, rather than a fundamental limitation of audio as a modality for cognitive-impairment detection.

External evaluation of the adaptation strategies on the DementiaBank Delaware dataset with a distinct population (MCI vs control) and different speech tasks supports the generalizability of the adaptation strategies. Using LLaMA 8B and GPT-4o, the adaptation strategies showed high performance in classifying MCI from control cases. ICL with the most-similar demonstration strategy provided a strong baseline, adding teacher-generated reasoning improved LLaMA 8B’s performance compared to ICL, and token-level fine-tuning resulted in the best overall performance. These findings also highlight the adaptation strategy’s effectiveness for early detection of MCI.

Our results suggest a simple decision framework for selecting adaptation strategies in future work. If fine-tuning is not feasible (eg, limited labeled data, limited computational resources), few-shot ICL can still perform strongly, especially when demonstrations are selected as average similarity to class centroids, the most consistent strategy across models. For smaller open-weight models in prompt-only configurations, structured reasoning approaches (eg, teacher-generated rationales or expert-grounded ToT prompting) improved performance under specific conditions, while self-consistency techniques reduced output variability. When labeled training data are available and on-premise computational constraints must be respected, parameter-efficient fine-tuning methods (eg, LoRA or quantized low-rank adaptation) provided the most reliable performance gains, achieving the highest overall accuracy among adaptation strategies. In settings where generative models showed instability in producing relevant tokens, reformulating the fine-tuning with a supervised classification head resulted in more stable and reproducible predictions. Finally, although task-specific fine-tuning improved multimodal audio-text models compared with zero-shot and in-context settings, these LLMs did not outperform the top-performing text-only models that were fine-tuned under the same data and evaluation mechanism. This indicates that, for this study’s task and dataset, incorporating acoustic features did not yield additional predictive gains beyond those captured in the transcripts.

### Comparison With Prior Work

The Pitt Corpus is a widely used benchmark for cognitive impairment detection from speech. Prior studies have used hand-crafted acoustic features (eg, Mel-Frequency Cepstral Coefficients), transformer-based embeddings (eg, Wav2Vec 2.0), rule-based linguistic metrics (eg, Linguistic Inquiry and Word Count), and BERT-based embeddings, achieving *F*_1_-scores between 0.70 and 0.87. Notable approaches include fine-tuning BERT-large with Automatic Speech Recognition scores [50] (*F*_1_=0.85), combining multiple BERT variants with support vector machine [38] (*F*_1_=0.85), and ensembling logistic regression with fine-tuned BERT or Enhanced Representation through Knowledge Integration [51] (*F*_1_=0.82). More recent work has leveraged coattention fusion [52] (*F*_1_=0.86), multimodal fusion with ChatGPT-derived embeddings [53] (*F*_1_=0.87), and cross-modal attention [54] (*F*_1_=0.84). In comparison, our LLM-based methods achieved an *F*_1_-score almost equal to 0.81 with ICL and 0.80‐0.83 with fine-tuning, demonstrating competitive performance with state-of-the-art systems.

These findings have important clinical implications. While biomarker-based tools (eg, blood pTau217 and β-amyloid assays [55]) offer diagnostic value, they do not capture functional changes in everyday communication. Language impairment, an early sign of cognitive decline, remains poorly integrated into current screening workflows. LLM-based speech analysis offers a scalable, noninvasive approach to detect linguistic changes that complement biological markers. Integrating these tools into clinical settings [56] could enable earlier detection, improve decision-making, and broaden access to timely care.

Future work should combine LLM-based speech analysis with biological data, evaluate performance across diverse populations to ensure fairness, and address implementation challenges such as clinician acceptance, workflow integration, and regulatory compliance. LLMs, particularly when adapted with representative examples and reasoning strategies, offer a promising foundation for scalable cognitive screening.

### Limitations

This study has several limitations. First, the use of 2 English datasets (the ADReSSo subset of DementiaBank Pitt Corpus and Delaware) limits generalizability to other speech corpora, languages, or dialects, potentially overlooking broader linguistic variability. Second, the limited training data, especially for multimodal models, may have restricted learning from acoustic inputs, making their underperformance difficult to interpret as model limitations alone. Third, reliance on automatic speech recognition (AWS) introduces transcription errors, particularly in impaired speech, which may disproportionately affect smaller models sensitive to input noise.

### Conclusion

This study provides the first systematic comparison of LLM-based adaptation strategies for detecting cognitive impairment from speech. Fine-tuned open-weight models matched or outperformed commercial LLMs and achieved performance comparable to advanced multimodal systems previously built on the benchmark Pitt Corpus. While current multimodal LLMs underperformed, results support LLM-based speech analysis as a scalable and effective approach for early cognitive screening.

## Supplementary material

10.2196/82608 Multimedia Appendix 1 In-context learning with demonstration selection prompt design.

10.2196/82608 Multimedia Appendix 2 Reasoning-based methods prompt design.

10.2196/82608 Multimedia Appendix 3 Tree-of-thought reasoning prompt design.

10.2196/82608 Multimedia Appendix 4 Fine-tuning prompt design.

10.2196/82608 Multimedia Appendix 5 Fine-tuning details and hyperparameters.

10.2196/82608 Multimedia Appendix 6 Token probability-based classification details.

10.2196/82608 Multimedia Appendix 7 Multimodal large language models fine-tuning prompt.

10.2196/82608 Multimedia Appendix 8 Multimodal large language models fine-tuning hyperparameters.

10.2196/82608 Multimedia Appendix 9 Definitions of linguistic measures.

## Abbreviations

ADRD: Alzheimer disease and related dementia
API: application programming interface
AUC: area under the curve
AWS: Amazon Web Service
BERT: Bidirectional Encoder Representations from Transformer
CN: cognitive normal
FN: false negative
FP: false positive
ICL: in-context learning
LLM: large language model
LoRA: Low-Rank Adaptation
MCI: mild cognitive impairment
MMSE: Mini-Mental State Examination
TN: true negative
TP: true positive
