# Supplementary material for: Large Language Model Adaptation Strategies in Speech-Based Cognitive Screening: Systematic Evaluation
Source: JMIR AI. 2026 Mar 26;5:e82608. doi: 10.2196/82608 (PMC13021110; doi:10.2196/82608)
Supplement: Multimedia Appendix 1 [file ai-v5-e82608-s001.docx]

To ensure consistency across all experiments in the few-shot setting, we employed a standardized prompt structure. The base prompt was as follows:


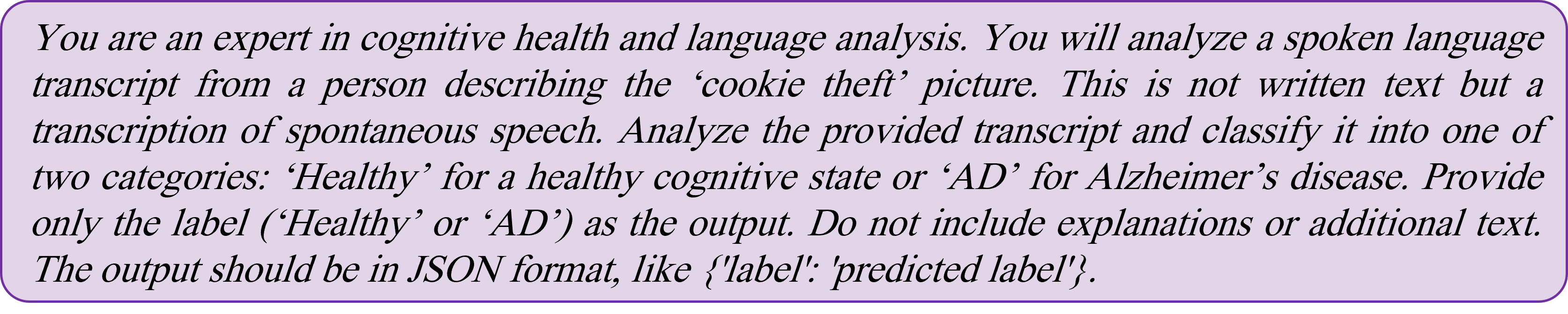


In the few-shot setting, demonstrations were introduced with the following prefix:


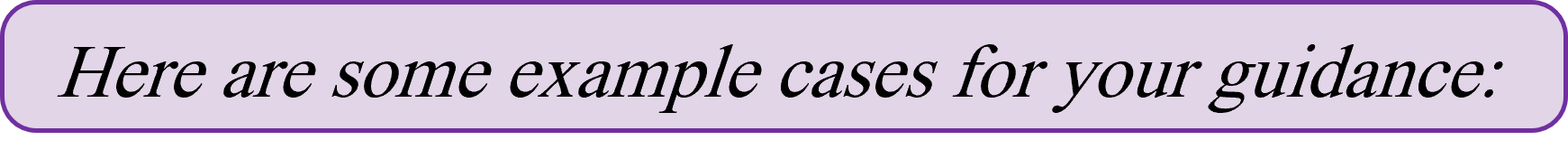


This ensured a consistent prompt structure, with demonstrations presented before the test input to guide the model’s prediction. Note that “Healthy” denotes cognitive normal and “AD” refers to cognitive impairment in the prompt.
