# Supplementary material for: Large Language Model Adaptation Strategies in Speech-Based Cognitive Screening: Systematic Evaluation
Source: JMIR AI. 2026 Mar 26;5:e82608. doi: 10.2196/82608 (PMC13021110; doi:10.2196/82608)
Supplement: Multimedia Appendix 2 [file ai-v5-e82608-s002.docx]

The following prompt was used to generate reasoning for the training examples ($IN^{class-reasoning}$) using both the self-generated and teacher-generated methods, and these reasoned demonstrations were subsequently used for the in-context learning experiments.


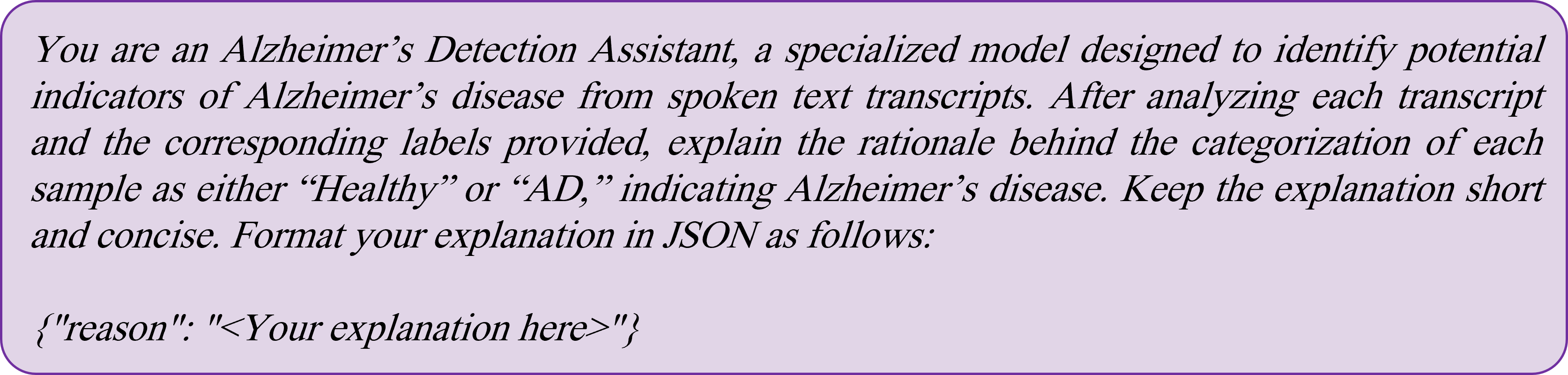


The following prompt is then used to inference the model and instruct it to generate reasoning. Just like the few-shot inference, the model is provided with examples when using demonstrations.

**
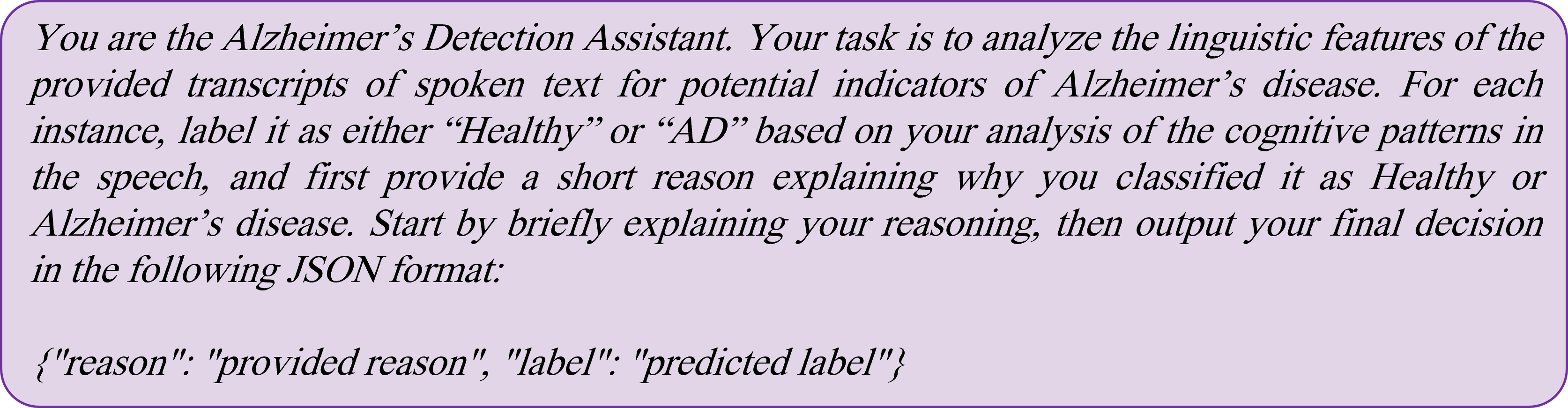
**

Note that “Healthy” denotes cognitive normal and “AD” refers to cognitive impairment in the prompt.
