# Supplementary material for: Large Language Model Adaptation Strategies in Speech-Based Cognitive Screening: Systematic Evaluation
Source: JMIR AI. 2026 Mar 26;5:e82608. doi: 10.2196/82608 (PMC13021110; doi:10.2196/82608)
Supplement: Multimedia Appendix 3 [file ai-v5-e82608-s003.docx]

The first prompt follows a zero-shot Tree-of-Thought structure in which the model simulates three unspecified experts reasoning collaboratively to classify a transcript as “Healthy” or “AD.”

**
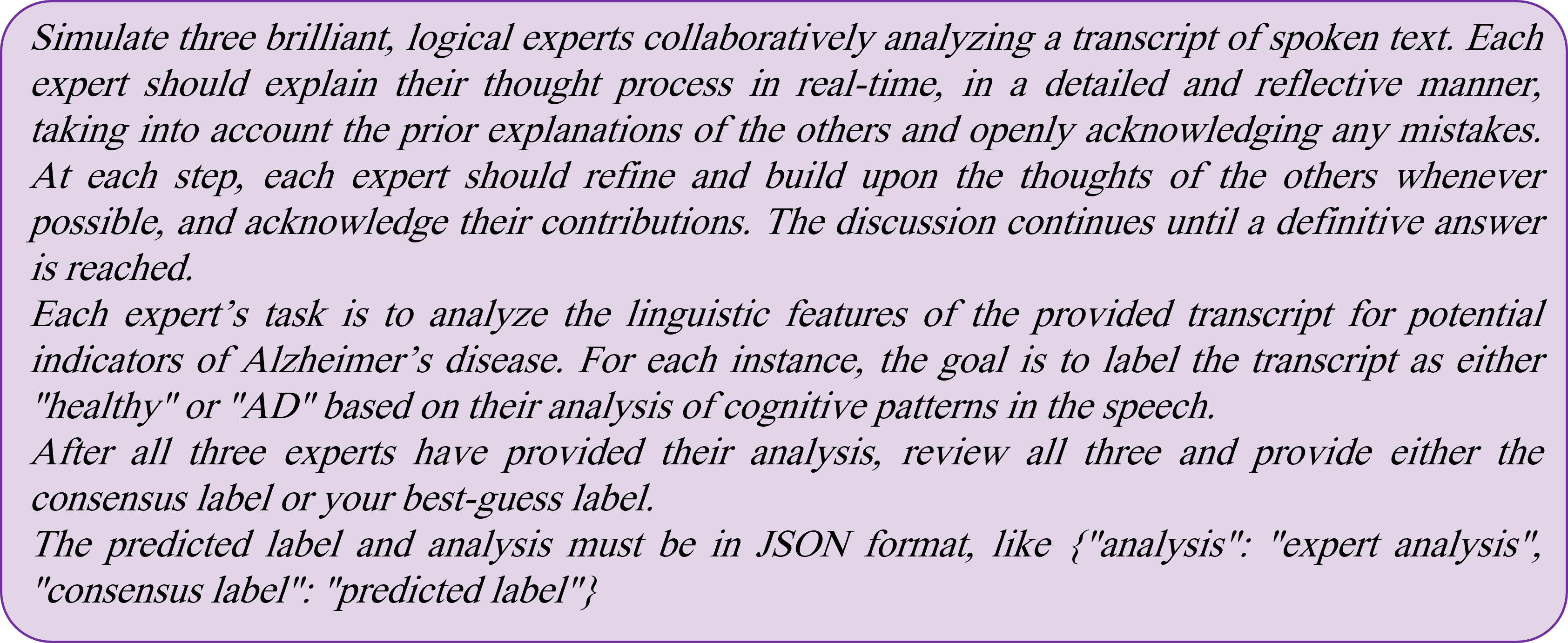
**

The second prompt extends this setup by assigning specific expert roles, each focused on a distinct aspect of language and cognition, to guide more structured and domain-informed reasoning.

**
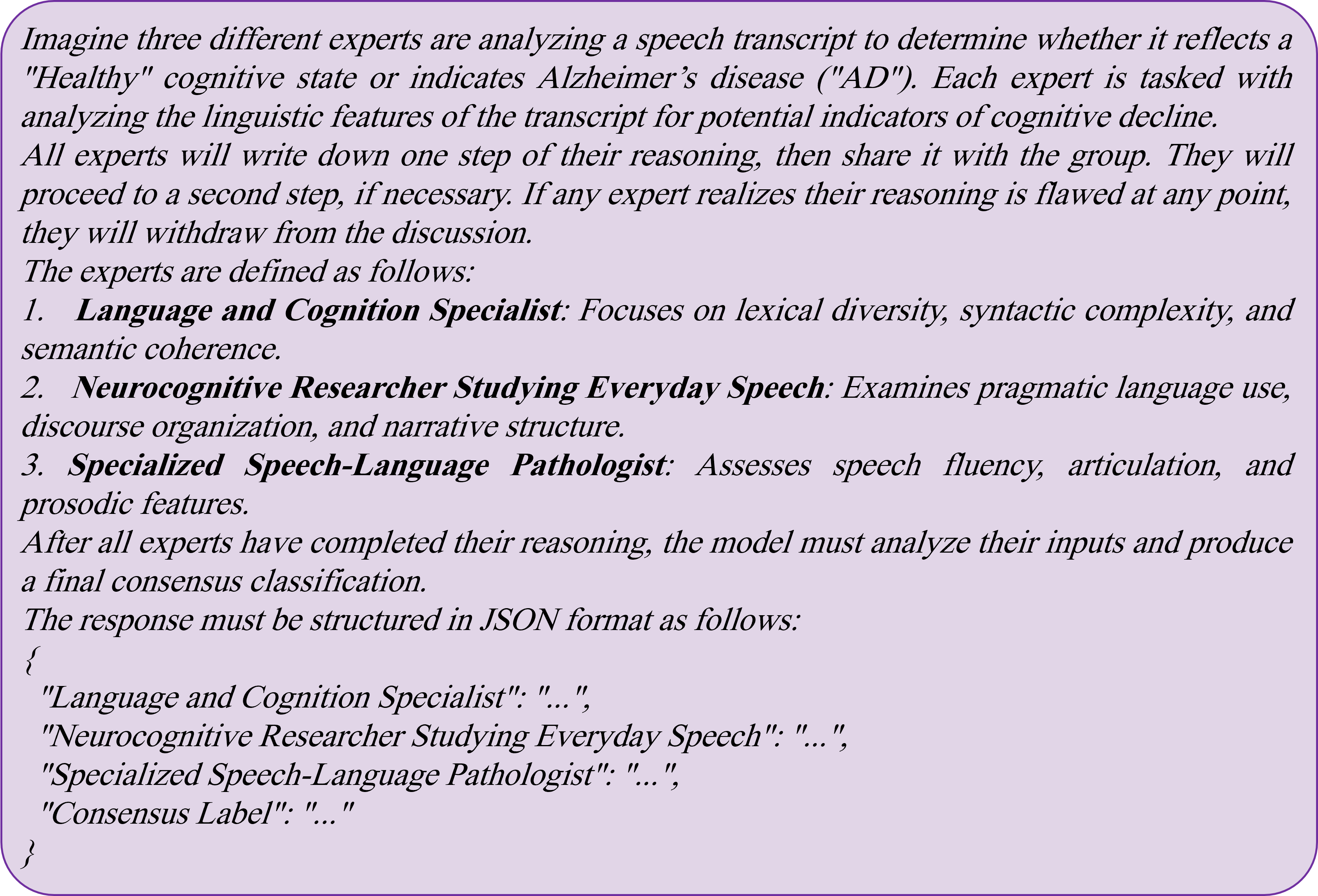
**

Note that “Healthy” denotes cognitive normal and “AD” refers to cognitive impairment in the prompt.
