# Supplementary material for: Large Language Model Adaptation Strategies in Speech-Based Cognitive Screening: Systematic Evaluation
Source: JMIR AI. 2026 Mar 26;5:e82608. doi: 10.2196/82608 (PMC13021110; doi:10.2196/82608)
Supplement: Multimedia Appendix 4 [file ai-v5-e82608-s004.docx]

The following prompt was used to fine-tune the models in both tuning approaches, text-generation and classification head, and was also used during inference to evaluate these fine-tuned models.


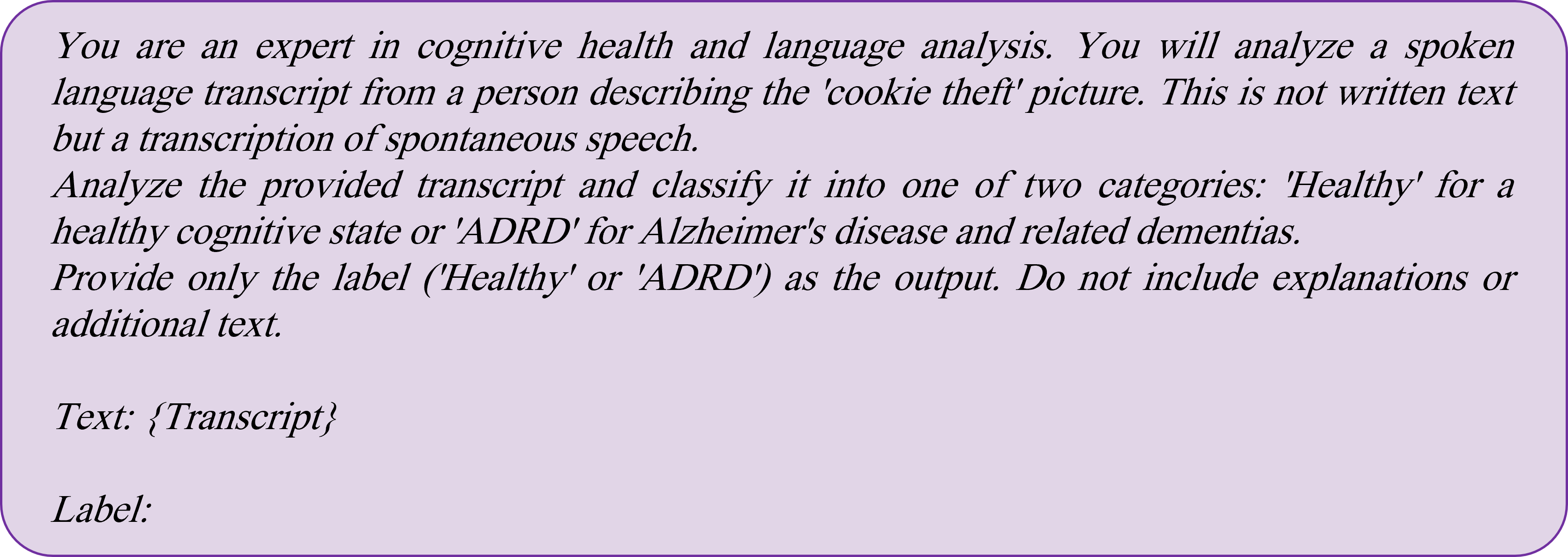


Note that “Healthy” denotes cognitive normal and “AD” refers to cognitive impairment in the prompt.
