# Supplementary material for: Large Language Model Adaptation Strategies in Speech-Based Cognitive Screening: Systematic Evaluation
Source: JMIR AI. 2026 Mar 26;5:e82608. doi: 10.2196/82608 (PMC13021110; doi:10.2196/82608)
Supplement: Multimedia Appendix 5 [file ai-v5-e82608-s005.docx]

This section provides the detailed settings used for fine-tuning the models on the Alzheimer's disease classification task using instruction-tuned text generation. The goal was to adapt each model to generate a binary label (“AD” or “Healthy”) in response to transcript classification prompts.

For open-weight models, we fine-tuned using QLoRA, enabling efficient adaptation with minimal computational overhead. The following hyperparameters were tuned:

- Quantization: 4-bit quantization applied to reduce memory footprint.
- Rank & Alpha: Low-rank adapter dimensions (ranks of 16–128), with alpha scaling (α = 2 × rank) to stabilize adapter outputs.
- Dropout: Regularization rates of 0, 0.05, and 0.10 explored to prevent overfitting.
- Learning Rate: Values of 2e-4 and 1e-4 tested, with 2e-4 yielding better convergence.
- Scheduler: Cosine annealing learning rate schedule to facilitate smooth convergence.
- Optimizer: PagedAdamW optimizer for efficient memory usage.
- Precision: Mixed-precision (FP16) training used across models.
- Batch Size & Gradient Accumulation: Adjusted based on model size to maintain effective batch sizes of 4–8.
- Epochs: Ranged from 1 to 13 depending on model size and validation performance.
- Max Sequence Length: Set to 1024 tokens to accommodate typical transcript lengths.

For API-based models (GPT-4o, Gemini-2.0), fine-tuning was limited to available parameters:

- Batch Size
- Number of Epochs
- Learning Rate Multiplier
- Adapter Size (for Gemini only)

The best performing parameters for each model are stated in the table below.

Table 2. Selected hyperparameters for LLM fine-tuning

| **Model** | **QLoRA Rank** | **QLoRA Alpha** | **QLoRA Dropout** | **Batch Size** | **Grad Accum** | **Epochs** | **LR Multiplier** | **Adapter Size** | **Hardware** |
| --- | --- | --- | --- | --- | --- | --- | --- | --- | --- |
| **LLaMA 3B** | 64 | 128 | 0.05 | 2 | 4 | 11 | - | - | NVIDIA A40 |
| **LLaMA 8B** | 64 | 128 | 0.1 | 4 | 2 | 12 | - | - | NVIDIA A40 |
| **MedAlpaca 7B** | 128 | 256 | 0.1 | 2 | 2 | 6 | - | - | NVIDIA A40 |
| **Ministral 8B (2410)** | 32 | 64 | 0 | 2 | 2 | 10 | - | - | NVIDIA A40 |
| **LLaMA 70B** | 16 | 32 | 0 | 1 | 4 | 9 | - | - | NVIDIA A100 |
| **GPT-4o (2024-08-06)** | - | - | - | 20 | - | 10 | 2.5 |  | API (OpenAI) |

To examine potential overfitting to the validation set and the sensitivity of model performance to hyperparameter choices, we report additional validation and test results in Tables 3–5.

Table 3 reports validation and test F1-scores for all token-based fine-tuned LLMs. Across models, validation performance is high and generally close to test performance. This similarity suggests that hyperparameter selection did not lead to overfitting to the 50-sample validation set.

Tables 4 and 5 examine the sensitivity of model performance to hyperparameter choices for LLaMA 3B and LLaMA 8B, the two top-performing token-level fine-tuned models. For each LoRA rank and dropout setting, the tables report the mean validation F1-score, the standard deviation of validation performance across runs, and the corresponding test F1-score. Across the hyperparameter values tested in our grid search, both validation and test F1-scores remain high and vary only slightly. These results indicate that model performance is not strongly sensitive to specific hyperparameter values when the search space is chosen based on the task, dataset size, and prior literature.

Model selection was performed using the validation set only, considering both validation F1-score and stability across runs. As a result, in some cases a configuration with slightly lower validation F1 but lower variability was selected over the single highest-scoring configuration with higher variance. All test set results were computed only after training and model selection were completed, and the test set was not used for hyperparameter tuning or selection.

Table 3. Validation and test F1-scores for fine-tuned text-based models.

| **Model** | **Validation F1-score** | **Test F1-score** |
| --- | --- | --- |
| **LLaMA 3B** | 0.83 | 0.83 |
| **LLaMA 8B** | 0.86 | 0.81 |
| **MedAlpaca 7B** | 0.81 | 0.78 |
| **Ministral 8B (2410)** | 0.80 | 0.77 |
| **LLaMA 70B** | 0.85 | 0.82 |
| **GPT-4o (2024-08-06)** | 0.74 | 0.79 |

Table 4. Ablation of QLoRA hyperparameters for LLaMA 3B.

| **QLoRA Rank** | **QLoRA Dropout** | **Validation F1-score** | **Validation STD** | **Test F1-score** |
| --- | --- | --- | --- | --- |
| 32 | 0.00 | 0.73 | 0.15 | 0.75 |
|  | 0.05 | 0.74 | 0.13 | 0.70 |
|  | 0.10 | 0.74 | 0.12 | 0.79 |
| 64 | 0.00 | 0.79 | 0.04 | 0.87 |
|  | **0.05** | **0.83** | **0.05** | **0.83** |
|  | 0.10 | 0.80 | 0.04 | 0.80 |
| 128 | 0.00 | 0.79 | 0.07 | 0.83 |
|  | 0.05 | 0.79 | 0.09 | 0.81 |
|  | 0.10 | 0.81 | 0.11 | 0.80 |

Table 5. Ablation of QLoRA hyperparameters for LLaMA 8B.

| **QLoRA Rank** | **QLoRA Dropout** | **Validation F1-score** | **Validation STD** | **Test F1-score** |
| --- | --- | --- | --- | --- |
| 32 | 0.00 | 0.82 | 0.04 | 0.78 |
|  | 0.05 | 0.82 | 0.12 | 0.79 |
|  | 0.10 | 0.74 | 0.07 | 0.82 |
| 64 | 0.00 | 0.81 | 0.05 | 0.79 |
|  | 0.05 | 0.86 | 0.24 | 0.83 |
|  | **0.10** | **0.86** | **0.06** | **0.81** |
| 128 | 0.00 | 0.81 | 0.04 | 0.85 |
|  | 0.05 | 0.81 | 0.24 | 0.85 |
|  | 0.10 | 0.84 | 0.13 | 0.83 |
