# Supplementary material for: Large Language Model Adaptation Strategies in Speech-Based Cognitive Screening: Systematic Evaluation
Source: JMIR AI. 2026 Mar 26;5:e82608. doi: 10.2196/82608 (PMC13021110; doi:10.2196/82608)
Supplement: Multimedia Appendix 6 [file ai-v5-e82608-s006.docx]

For this method, models were fine-tuned using next-token prediction loss. During inference, the logits corresponding to the target tokens “AD” and “Healthy” were extracted from the model’s output layer. The probabilities were computed using softmax:

$$P(Label) =\frac{e^{logit_{label}}}{e^{logit_{AD}}+e^{logit_{Healthy}}}$$

The final label was assigned as:

$$L_{s} = argmax(P(AD), P(Healthy))$$

For commercial models using the *top_logprobs* API, if either token was missing from the top 5, its probability was estimated as:

$$P_{missing} = 1-\sum_{top 5} P_{i}$$

Where $P_{i}$ is the probability for token $i$ in the top five *logprobs* returned.

This ensured consistent label determination across models with limited output token visibility.
