# Supplementary material for: Large Language Model Adaptation Strategies in Speech-Based Cognitive Screening: Systematic Evaluation
Source: JMIR AI. 2026 Mar 26;5:e82608. doi: 10.2196/82608 (PMC13021110; doi:10.2196/82608)
Supplement: Multimedia Appendix 7 [file ai-v5-e82608-s007.docx]

The following prompt yielded the best performance when applied to the fine-tuned models, Phi 4 Multimodal and Qwen 2.5 Omni. The same prompt was used during both training and inference.


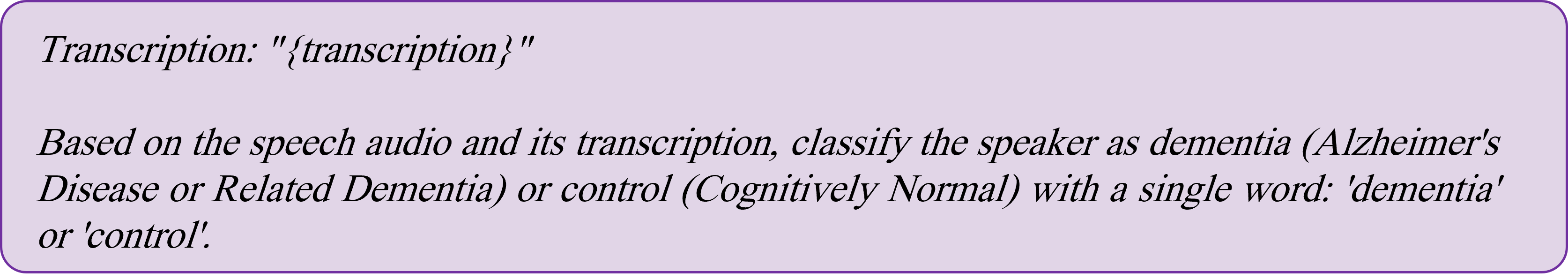


Note that “control” denotes cognitively normal and “dementia” refers to cognitive impairment in the prompt.
