# Supplementary material for: Large Language Model Adaptation Strategies in Speech-Based Cognitive Screening: Systematic Evaluation
Source: JMIR AI. 2026 Mar 26;5:e82608. doi: 10.2196/82608 (PMC13021110; doi:10.2196/82608)
Supplement: Multimedia Appendix 8 [file ai-v5-e82608-s008.docx]

The following hyperparameter values were used to fine-tune the two multimodal models.

Table 6. Multi-modal LLMs tuning Hyperparameters

| **Model** | **LoRA Rank** | **Epochs** | **Batch Size** | **Gradient Accumulation Step** | **Learning Rate** | **Maximum Audio Length (seconds)** | **LR Scheduler** | **Warmup Ratio** |
| --- | --- | --- | --- | --- | --- | --- | --- | --- |
| **Phi-4** | 320 | 3 | 1 | 32 | 2e-5 | 70 | — | — |
| **Qwen-2.5-Omni** | 64 | 5.0 | 1 | 4 | 1.0e-4 | — | cosine | 0.1 |

For LoRA adaptation and fine-tuning, we used all self-attention and MLP (feed-forward) blocks in Phi-4’s language decoder and all transformer layers in Qwen-2.5-Omni, following the layer selections specified in the models’ original papers^31,32^.

For Phi-4 Multimodal, we systematically examined the effect of audio segment length during fine-tuning by evaluating maximum durations from 40 to 120 seconds in 10-second increments. For a given maximum length L, recordings longer than L were truncated to the first L seconds, whereas shorter recordings were retained in full; the same procedure was applied consistently across the fine-tuning, validation, and test sets. Figure A1 reports the F1-score for the cognitive-impairment class on the test set as a function of audio duration. Performance increased steadily as segment length expanded from 40 to 70 seconds, indicating that shorter clips do not provide sufficient speech to reliably capture cognitive-impairment–related cues. Beyond 70 seconds, performance fluctuated without a consistent upward trend, suggesting diminishing returns from additional audio input. Overall, these results indicate a trade-off between including enough speech to capture diagnostically relevant speech clues and incorporating additional material that does not consistently improve performance. Performance peaked at 70 seconds, which provided sufficient linguistic content for effective fine-tuning, whereas longer segments introduced greater variability without systematic gains, likely due to less informative speech and a reduced effective sample size at longer durations. Based on this analysis, 70 seconds was selected as the optimal audio length for Phi-4.


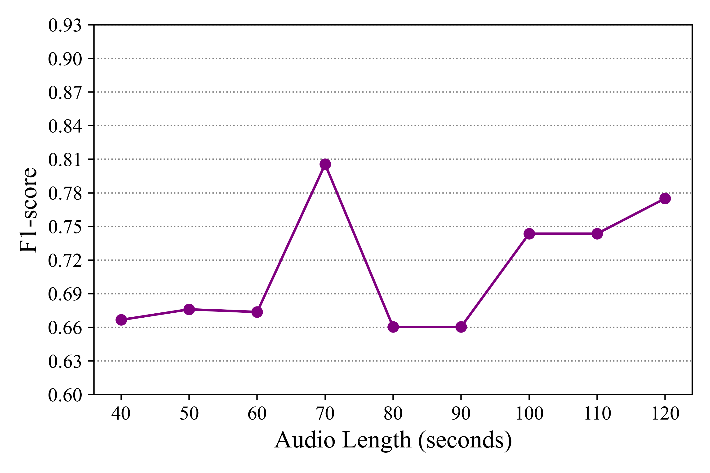


Figure A 1. F1-score (cognitively impaired class) for Phi-4 Multimodal on the test set when fine-tuned with different maximum audio lengths (40–120 s). Performance peaks at 70 s, which we therefore use in the main experiments.
