# Supplementary material for: Large Language Model Adaptation Strategies in Speech-Based Cognitive Screening: Systematic Evaluation
Source: JMIR AI. 2026 Mar 26;5:e82608. doi: 10.2196/82608 (PMC13021110; doi:10.2196/82608)
Supplement: Multimedia Appendix 9 [file ai-v5-e82608-s009.docx]

This appendix defines the linguistic measures used to characterize Cookie Theft transcripts in our error analysis. Measures are grouped into Lexical Richness, Syntactic Complexity, Disfluencies and Repetition, and Semantic Coherence/Referential Clarity, and were computed from text to compare correctly (TP/TN) versus incorrectly (FP/FN) classified cases. Table below depicts a brief explanation of the features calculated.

Table 7. Textual features calculated for quantitative error analysis.

| **Category** | **Feature** | **Description** |
| --- | --- | --- |
| **Lexical Richness** | Type–Token Ratio (TTR) | Proportion of unique words to total words, indicating vocabulary diversity. |
|  | Root Type–Token Ratio (RTTR / Guiraud’s) | Length-adjusted vocabulary diversity using unique words scaled by text length. |
|  | Corrected Type–Token Ratio (CTTR / Carroll’s) | Length-adjusted ratio of unique to total words to reduce text-length bias. |
|  | Brunet’s Index | Vocabulary diversity measure less sensitive to text length; lower values indicate richer vocabulary. |
|  | Honoré’s Statistic | Emphasizes rare words (hapax legomena) to capture lexical richness |
|  | Measure of Textual Lexical Diversity | Assesses how consistently lexical diversity is maintained across the text. |
|  | Hypergeometric Distribution Diversity | Probability-based estimate of lexical diversity accounting for sampling effects. |
|  | Unique/Total Word Ratio | Fraction of unique words among all words, reflecting repetition vs. variety. |
|  | Unique Word Count | Number of distinct words used in the text. |
|  | Lexical Frequency | Average commonness of words based on a reference lexicon; higher values indicate more frequent words. |
|  | Content Words Ratio | Proportion of nouns, verbs, adjectives, and adverbs, indicating information density. |
| **Syntactic Complexity** | Part-of-Speech Rate | Distribution of major POS categories, indexing grammatical variety. |
|  | Relative Pronouns Rate | Proportion of relative pronouns (e.g., who, which, that), indicating use of subordinate relative clauses. |
|  | Determiners Ratio | Proportion of determiners, reflecting specificity and clarity of reference. |
|  | Verbs Ratio | Proportion of verbs, indexing predicate density and event description. |
|  | Nouns Ratio | Proportion of nouns, indexing information density and entity mention. |
|  | Negative Adverbs Rate | Proportion of negative adverbs, reflecting use of negation and more complex expression. |
|  | Word Count | Total number of words as a proxy for elaboration and planning. |
| **Disfluencies and Repetition** | Speech Rate | Tempo of speech, indexing fluency. |
|  | Consecutive Repeated Clauses Count | Number of back-to-back repeated phrases or clauses, indexing perseveration. |
| **Semantic Coherence** | Content Density | Amount of meaning-bearing content relative to text length. |
|  | Reference Rate to Reality | Frequency of concrete references grounded in the picture or real-world entities/events. |
|  | Pronouns Ratio | Proportion of pronouns, which can reduce clarity when antecedents are unclear. |
|  | Definite Articles Ratio | Proportion of definite articles, indicating reference to specific, known entities. |
|  | Indefinite Articles Ratio | Proportion of indefinite articles, indicating introduction of new or nonspecific entities. |
